# Arrays of Ag-nanoparticles decorated TiO_2_ nanotubes as reusable three-dimensional surface-enhanced Raman scattering substrates for molecule detection

**DOI:** 10.3389/fchem.2022.992236

**Published:** 2022-10-03

**Authors:** Haichao Zhai, Chuhong Zhu, Xiujuan Wang, Yupeng Yuan, Haibin Tang

**Affiliations:** ^1^ College of Chemistry and Chemical Engineering, School of Materials Science and Engineering, Anhui University, Hefei, China; ^2^ School of Microelectronics, Hefei University of Technology, Hefei, China; ^3^ Key Laboratory of Materials Physics, Anhui Key Laboratory of Nanomaterials and Nanotechnology, Institute of Solid State Physics, HFIPS, Chinese Academy of Sciences, Hefei, China

**Keywords:** surface enhanced Raman scattering, silver nanoparticle, TiO_2_ nanotube, detection, self-cleaning

## Abstract

Three-dimensional surface-enhanced Raman scattering (SERS) substrates usually provide more hot spots in the excitation light beam and higher sensitivity when compared with the two-dimensional counterpart. Here a simple approach is presented for the fabrication of arrays of Ag-nanoparticles decorated TiO_2_ nanotubes. Arrays of ZnO nanorods were fabricated in advance by a hydrothermal method. Then TiO_2_ nanotube arrays were achieved by immersing the arrays of ZnO nanorods in an aqueous solution of (NH_4_)_2_TiF_6_ for 1.5 h. Vertically aligned TiO_2_ nanotube arrays were modified with dense Ag nanoparticles by Ag mirror reaction. High density of Ag nanoparticles decorated on the fabricated TiO_2_ nanotubes provide plenty of hotspots for Raman enhancement. In addition, the fabricated array of Ag nanoparticles modified TiO_2_ nanotubes can serve as a reusable SERS substrate because of the photocatalytic activity of the TiO_2_ nanotubes. The SERS substrate adsorbed with analyte molecules can realize self-cleaning in deionized water after UV irradiation for 2.5 h. The sensitivity of the fabricated SERS substrate was investigated by the detection of organic dye molecules. The detectable concentration limits of rhodamine 6G (R6G), malachite green (MG) and methylene blue (MB) were found to be 10^−12^ M, 10^−9^ M and 10^−8^ M, respectively. The enhancement factor (EF) of the three-dimensional SERS substrate was estimated to be as high as ∼1.4×10^8^. Therefore, the prepared Ag nanoparticles modified TiO_2_ nanotube arrays have promising potentials to be applied to rapid and trace SERS detection of organic chemicals.

## Introduction

Organic dyes such as rhodamine 6G (R6G), malachite green (MG), and methylene blue (MB) are widely used in the paper industry, aquaculture, textiles, cosmetics and so on (Biparva et al., 2010; Sivashanmugan et al., 2015; Kumar et al., 2017; Jia et al., 2018; Chao et al., 2020; Zhang et al., 2022). While they provide convenience for human production and life, the dye residue-induced damage is also coming quietly. For example, excessive R6G in cosmetics not only could cause irritation to the skin and eyes, but also could induce toxicity and carcinogenicity to human beings (Biparva et al., 2010; Ranjbari and Hadjmohammadi, 2015). MG and MB can improve the survival rate of aquaculture and resist the infection of fish by various bacteria, fungi and parasites (Sivashanmugan et al., 2015; Zhang et al., 2022). However, MG has mutagenic and teratogenic effects on humans, with mutagenicity, genotoxicity and carcinogenicity (Sivashanmugan et al., 2015; Kumar et al., 2017). MB is toxic to nerve tissue, reproductive system and skin. Accidental large intake of MG or MB will lead to chest pain, severe headache, mental confusion, urination pain and other symptoms (Ghosh and Bhattacharyya, 2002; Jia et al., 2018; Xu et al., 2021). Therefore, it is inevitable to develop an efficient, sensitive and economical method for the sensitive and rapid detection of organic dyes.

In comparison to traditional technologies such as infrared (IR) spectroscopy and gas chromatography (GC) (Song et al., 2016; Li and Chin, 2021), surface-enhanced Raman scattering (SERS) spectroscopy has lower cost and comparable or even more superior sensitivity. Therefore, SERS technology has bright prospects in many fields, such as trace detection, environmental monitoring, food safety, biochemical sensing and so on (Zhu et al., 2021a; Li et al., 2021; Yan et al., 2021). SERS as a powerful technology can detect trace molecules in a fast, sensitive and non-destructive way and provide molecular “fingerprint” information. At the same time, compared with IR spectroscopy, SERS also has the advantage of resisting interference from water (Zhou et al., 2018; Wen et al., 2021; Zhang et al., 2022). Under optimized conditions, even single molecule detection can be achieved (Kasztelan et al., 2021).

The enhancement of Raman scattering by noble metal nanostructures is dominated by the electromagnetic enhancement mechanism, which involves both the enhancement of the incident excitation and that of the scattered Raman fields and is a major contributor to SERS enhancements (Kumar et al., 2020a). Compared with other noble metals, silver (Ag) has the highest enhancement factor for the identical nanostructures due to its special dielectric function at the excitation wavelength range (Le Ru and Etchegoin, 2008; Zhu et al., 2021b). Semiconductor materials not only have high optical stability, but also have certain SERS activity and reusability (Wang and Guo, 2020; Wang et al., 2022). In addition, the interfacial charge transfer process of semiconductor materials can improve the polarization tensor of the target molecules, so as to enhance the vibrational scattering of the molecules. Therefore, loading plasmonic nanoparticles on the surface of semiconductor materials can not only improve the sensitivity of SERS detection by changing the electron cloud density on both metal and semiconductor materials and the charge transfer between semiconductor surface and target molecules (Wang et al., 2022), but also realize the reuse of the SERS substrate. As a common semiconductor material, nanostructured TiO_2_ has excellent photocatalytic performance, chemical stability and nontoxicity, so it can degrade organic molecules adsorbed on its surface and thus realize self-cleaning (Li et al., 2010; Kochuveedu et al., 2012). Therefore, the combination of TiO_2_ nanomaterials with Ag nanoparticles could obtain highly sensitive and recyclable SERS substrates.

**FIGURE 1:**
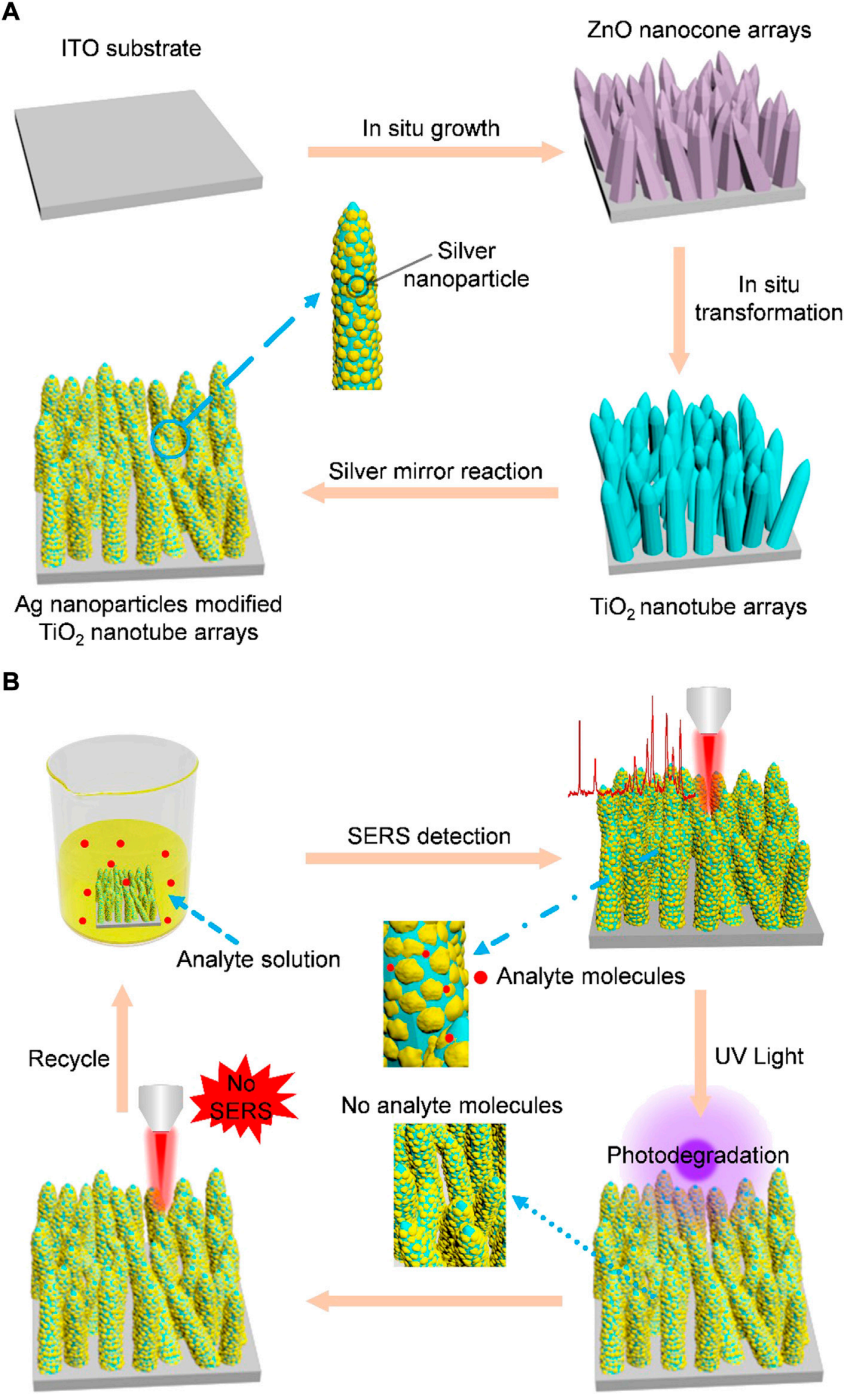
**(A)** Schematic for the synthesis of Ag nanoparticles modified TiO_2_ nanotube arrays. Step 1: ZnO nanocone arrays are grown on an ITO glass by a hydrothermal synthesis method. Step 2: TiO_2_ nanotube arrays are fabricated by immersing the as-prepared ZnO nanocones (serving as sacrificial templates) in an aqueous solution of (NH_4_)_2_TiF_6_. The hydrolysis reaction-produced TiO_2_ is deposited on the surface of ZnO nanocones and ZnO nanocone template is selectively etched by H^+^ gradually in the same time, leading to the final formation of TiO_2_ nanotubes. Step 3: Ag nanoparticles are modified on TiO_2_ nanotubes by silver mirror reaction. **(B)** Schematic for the self-cleaning performance of Ag nanoparticles modified TiO_2_ nanotube arrays. Step 1: Analyte molecules adsorb on the fabricated SERS and SERS detection are realized. Step 2: After realization of SERS detection, the analyte molecules adsorbed on the SERS substrate are removed by photodegradation. Step 3: SERS measurement is used to prove that the analyte molecules have been removed. Step 4: The recycled SERS substrate is used for detection of molecules again.

Compared with the two-dimensional counterpart, three-dimensional SERS substrates can provide larger surface area, more hotspots in the excitation light beam, and thus higher sensitivity (Li et al., 2018; Pal et al., 2019; Tsao et al., 2021). Therefore, three-dimensional nanoporous Al-Ag zig-zag silver nanorod arrays and buckled PDMS silver nanorod arrays fabricated by glancing angle deposition (GLAD), and three-dimensional hybrid MoS_2_/AgNPs/inverted pyramid PMMA resonant cavity system have shown excellent SERS performance (Kumar et al., 2015; Rajput et al., 2017; Li et al., 2018). Here, we propose a simple strategy to synthesize Ag nanoparticles modified TiO_2_ nanotube arrays. As shown in Figure 1A, ZnO nanocone arrays were firstly grown on an ITO substrate by water bath growth method, and then the fabricated ZnO nanocones were transformed into TiO_2_ nanotube arrays *in situ* by immersing the ZnO nanocone arrays in ammonium fluorotitanate solution. Finally, Ag nanoparticles were modified on the surfaces of TiO_2_ nanotubes by silver mirror reaction. The prepared Ag nanoparticles modified TiO_2_ nanotube arrays are homogeneously distributed in a large area. The high-density Ag nanoparticles on the surface of TiO_2_ nanotubes produce a large number of SERS hotspots. Therefore, the fabricated SERS substrates have good signal reproducibility and high SERS sensitivity. The SERS substrate can detect R6G, MG and MB with low detectable concentration limits of 10^−12^ M, 10^−9^ M and 10^−8^ M respectively. In addition, the fabricated Ag nanoparticles modified TiO_2_ nanotube arrays also showed good self-cleaning performance. As shown in Figure 1B, after irradiation with UV light, the Ag nanoparticles modified TiO_2_ nanotube arrays can degrade the analyte molecules adsorbed on the substrate, realizing the reuse of SERS substrates, and greatly saving the cost. Besides, the fabricated SERS substrates have good chemical stability in both acidic and alkaline solutions, allowing them to be used in a wide range of environments.

## Experimental section

### Chemicals

Zinc nitrate (Zn(NO_3_)_2_), ammonia water (NH_3_•H_2_O), and glucose (C_6_H_12_O_6_•H_2_O) were purchased from Sinopharm Chemical Reagent Co., Ltd. Ammonium hexafluorotitanate ((NH_4_)_2_TiF_6_), boric acid (H_3_BO_3_) and Ag nitrate (AgNO_3_) were obtained from Aladdin Chemicals. Indium tin oxide (ITO) glasses were bought from Huananxiangcheng Technology Co., Ltd (Shenzhen, China). Deionized water (18.25 MΩ cm^−1^) was obtained from a Millipore water purification. All chemicals were used without further purification.

### Growth of ZnO nanocone arrays on an ITO glass

ITO glasses (4 cm × 0.5 cm) were rinsed successively by deionized water, acetone, and ethanol in an ultrasonic cleaner. Then ITO glasses were further cleaned by plasma (PDC-32G) for 10 min to increase the surface hydrophilicity. After plasma treatment, the ITO glasses were vertically immersed in an aqueous solution of Zn(NH_3_)_4_(NO_3_)_2_ (100 ml, 0.1 M) under 80°C for 1 h. Then the ITO glass coated with vertically aligned ZnO nanorod array was taken out, rinsed with deionized water, and dried by flowing Ar gas.

### Fabrication of TiO_2_ nanotube arrays

Vertically aligned TiO_2_ nanotube arrays on an ITO glass were fabricated by immersing the fabricated ZnO nanorods arrays in an aqueous mixture of 0.075 M (NH_4_)_2_TiF_6_ and 0.2 M H_3_BO_3_ at room temperature for 1.5 h (Li et al., 2010). Then TiO_2_ was deposited on the surface of ZnO nanorods due to the hydrolysis of ammonium hexafluorotitanate in water. Meanwhile, ZnO was dissolved slowly by H^+^ ions produced by the hydrolysis of ammonium hexafluorotitanate. Therefore, TiO_2_ nanotube arrays on the ITO glass were finally obtained. The ITO glass coated with TiO_2_ nanotubes arrays was taken out, rinsed with deionized water for several times, and then dried by flowing Ar gas.

### Modification of Ag nanoparticles on TiO_2_ nanotubes

First, in order to prepare an Ag ammonia solution, a diluted 10 wt% NH_3_·H_2_O solution was gradually dropped into an aqueous solution of AgNO_3_ (20 mM, 100 ml) until the solution became clear again. Then 50 mmol glucose was added into the Ag ammonia solution under continuous stirring for 10 min. After that, the mixture solution was placed in a water bath (50°C), and the ITO glass with TiO_2_ nanotube arrays were immersed in the mixture solution for 25 min to modify Ag nanoparticles on TiO_2_ nanotubes through reduction reaction. After Ag nanoparticle decoration, the ITO glass coated with Ag nanoparticles modified TiO_2_ nanotube arrays was taken out and rinsed with deionized water for several times, and then dried by flowing Ar gas.

### SERS sample preparation

For SERS measurement, a SERS substrate (0.5 cm × 0.5 cm) was immersed in an aqueous solution of R6G, MG or MB with various concentrations for 3 h. Then the substrate adsorbed with analyte molecules was taken out and dried using flowing Ar gas. After SERS detection, UV light (365 nm, 220 mW cm^−2^) was employed to photodegradation of analyte molecules adsorbed on the SERS substrate. The adsorption of analyte molecules, SERS detection and photodegradation recycle was repeated for more than 3 times.

### Characterization

The fabricated Ag nanoparticles modified TiO_2_ nanotube arrays were characterized by a scanning electron microscopy (SEM, Hitachi Regulus 8230) and a transmission electron microscopy (TEM, JEOL JEM-2100). The optical absorption spectrum was measured by a UV−vis spectrophotometer (Hitachi, U-4100). The crystal structures were characterized using an X-ray diffractometer (XRD, Rigaku SmartLab 9 kW). SERS measurements were conducted using a confocal microprobe Raman system (Renishaw, inVia-Reflex) with a 532 nm excitation laser. In SERS measurements, the laser beam was vertically incident on the samples through a ×50 objective. The laser energy power reaching the sample surface was ∼0.5 mW. The integration duration of SERS spectra was 10 s. The diameter of the laser beam spot on samples was ∼5 μm.

## Results and discussion

**FIGURE 2:**
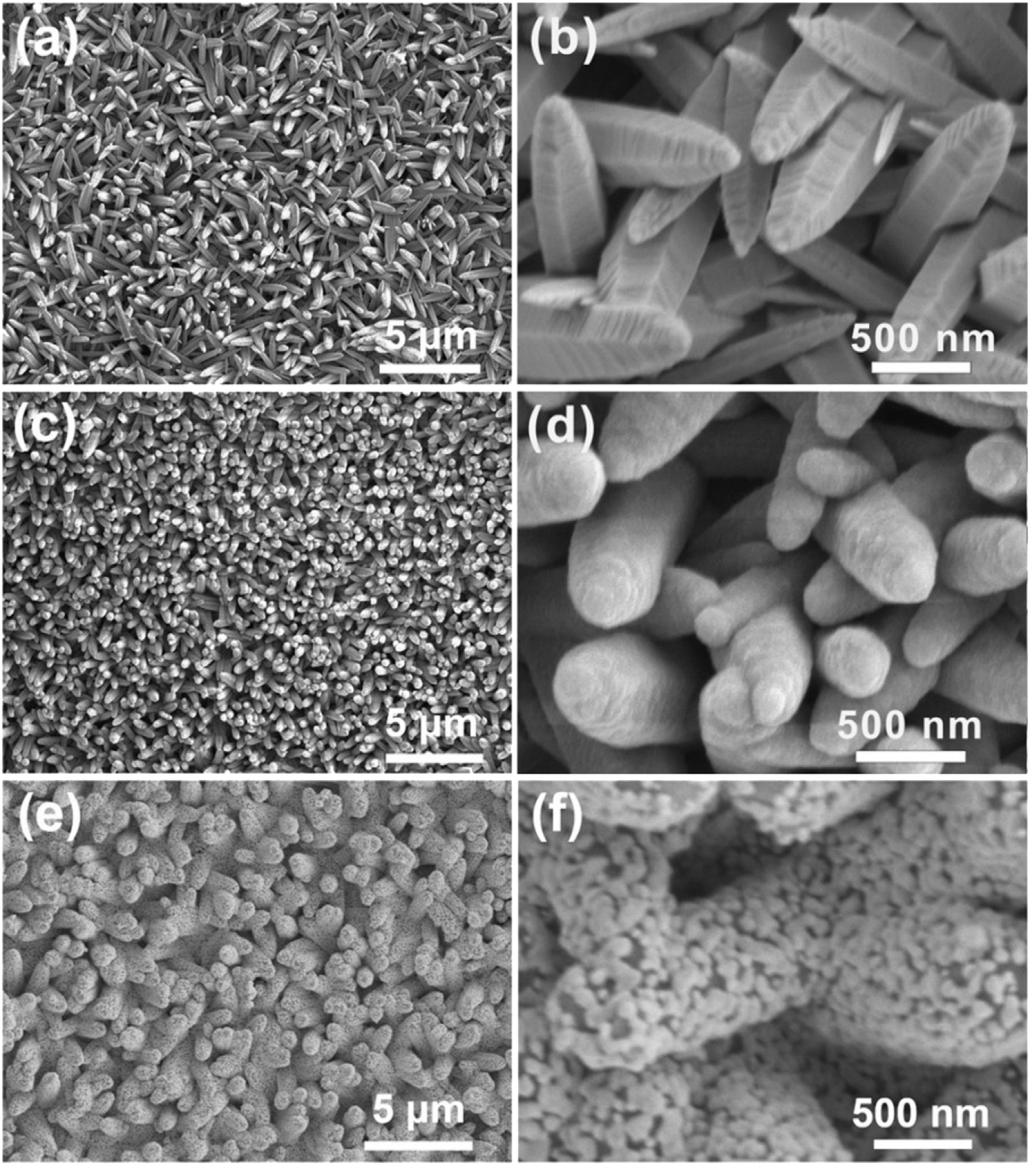
Morphology characterization. **(A)** SEM image of ZnO nanocone arrays. **(B)** An enlarged view of **(A)**. **(C)** SEM image of TiO_2_ nanotube arrays. **(D)** An enlarged view of **(C)**. **(E)** SEM image of Ag nanoparticles modified TiO_2_ nanotube arrays. **(F)** An enlarged view of **(E)**.

The micro-structures of nanomaterials are crucial to SERS activity, so the structures and morphologies of the fabricated vertically aligned ZnO nanocones, top-closed TiO_2_ nanotubes, and Ag nanoparticles modified TiO_2_ nanotube arrays were firstly characterized using an SEM. It can be clearly observed that ZnO nanocones grow vertically and uniformly on an ITO glasse (Figures 2A,B). The fabricated ZnO nanocone arrays can serve as sacrificial templates for *in situ* formation of TiO_2_ nanotube arrays. It was found that ZnO nanocones could be completely transformed into TiO_2_ nanotubes with similar appearance through TiCl_4_ treatment (Figures 2C,D). The fabricated TiO_2_ nanotubes with a top-closed, hollow tubular geometric structure, are vertically grown on the ITO substrate (Figures 2C,D, Supplementary Figure S1). The average diameter, average length, and average thickness of the TiO_2_ nanotubes are ∼500 nm, ∼ 2 µm and ∼100 nm, respectively. During the process of transforming ZnO nanocones into TiO_2_ nanotubes, the following chemical reactions were occurring:

$$\begin{array}{c} {TiF}_{6}^{2-}+2H_{2}O=TiO_{2}+6F^{-}+4H^{+} \end{array}$$ (1)

$$H_{3}{BO}_{3}+4HF=HBF_{4}+3H_{2}O$$ (2)

$$ZnO+2H^{+}=Zn^{2+}+H_{2}O$$ (3)

After ZnO nanocones being immersed in the reaction solution, the mixed solution of (NH_4_)_2_TiF_6_ and H_3_BO_3_ generated TiO_2_ and H^+^ through hydrolysis reaction. TiO_2_ was deposited on the surfaces of ZnO nanocones to form TiO_2_ nanotubes. During the formation of TiO_2_ nanotubes, the ZnO nanocone template was also selectively etched by H^+^ gradually. When the ZnO nanocones were completely etched, TiO_2_ nanotubes with hollow structure were achieved (Lee et al., 2005; Li et al., 2010). It was found that the ZnO nanocones were completely converted to TiO_2_ nanotubes because there were no characteristic peaks for Zn^2+^ in the energy dispersive spectrometry (EDS) spectrum of the fabricated TiO_2_ nanotube arrays on an ITO glass (Supplementary Figure S2). The ion exchange reaction between Zn^2+^ and Ti^4+^ can be explained based on hard–soft acid–base theory. Therefore, Ti^4+^ cations bind strongly with the O^2-^ anions to form TiO_2_ since Ti^4+^ is a harder acid than Zn^2+^ (Muduli et al., 2011). In comparison to ZnO nanocone, TiO_2_ nanotube has higher photocatalytic efficiency and better corrosion resistance (Chakraborty et al., 2010; Li et al., 2010). The vertically aligned TiO_2_ nanocone arrays provide a three-dimensional framework for the construction of three-dimensional SERS substrate. In order to achieve high SERS activity, we tried to decorate high-density Ag nanoparticles on the surfaces of the fabricated TiO_2_ nanotubes. It is found that Ag nanoparticles were densely and uniformly decorated on the surfaces of TiO_2_ nanotubes by silver mirror reaction (Figures 2E,F). Such Ag particles with an average size of ∼60 nm connect with each other to form an island-like film attached on the TiO_2_ nanotubes. There are a large number of nano-gaps with width of ≤10 nm in the island-like Ag nanofilm, providing high-density three-dimensionally distributed hotspots for Raman enhancement. The Ag nanoparticles are densely modified on TiO_2_ nanotubes to form island-like nanofilm (Supplementary Figure S3, S4). The Ag nanoparticles are firmly attached on the surface of TiO_2_ nanotube (Supplementary Figure S3B). The EDS mapping was conducted to confirm the element distributions of Ag nanoparticles modified TiO_2_ nanotube arrays. The EDS mapping results represent that the Ag nanoparticles are uniformly distributed on the surfaces of TiO_2_ nanotube (Supplementary Figure S5). Then X-ray diffraction (XRD) measurement was performed to study the crystal structure of the sample. The peaks around 37.9^°^, 44.1^°^, 64.3^°^, 77.4^°^, 81.5^°^ are indexed to the (111), (200), (220), (311) and (222) crystalline planes of Ag nanoparticles, respectively. Because TiO_2_ nanotubes present an amorphous state of TiO_2_, the TiO_2_ peaks disappear in the XRD spectrum (Supplementary Figure S6). The UV-vis spectrum of the Ag nanoparticles modified TiO_2_ nanotube arrays demonstrates that there is an absorption band from 340 to 550 nm (Supplementary Figure S7). Therefore, to excite the LSPR mode, the excitation laser of 532 nm is used to measure the SERS activity in the following experiments.

Obviously, the size of Ag nanoparticles could be regulated by the concentration of AgNO_3_ and the reaction duration. As shown in Supplementary Figure S8, when the concentration of AgNO_3_ increase from 5 to 50 mM, the size and spatial distributed density of Ag nanoparticles are increasing accordingly. When the AgNO_3_ concentration reaches 50 mM, Ag nanoparticles grow big and connect with each other, leading to the disappearance of some nano-gaps between neighboring Ag nanoparticles. Therefore, high concentration of AgNO_3_ (≥50 mM) could induce the reduction of the number of hotspots and the decrease of SERS activity. Similarly, when prolonging the silver mirror reaction duration from 5 to 50 min (Supplementary Figure S9), the size of Ag nanoparticles becomes bigger and bigger; the density of Ag nanoparticles increases accordingly. Therefore, Ag nanoparticles would be assembled to form shells with a few pores coating on the TiO_2_ nanotubes under long silver mirror reaction duration (Supplementary Figure S8D). There is an optimized experimental condition for high SERS activity. To obtain the optimized SERS performance, Raman measurement was conducted using different samples as SERS substrates. It was found that the SERS signals changed obviously when tuning the concentrations of AgNO_3_ and keeping the silver mirror reaction duration unchanged. The highest SERS activity is achieved when the concentration of AgNO_3_ is 20 mmol/L (Supplementary Figure S10). Similarly, the intensity of Raman bands reaches the maximum value when the silver mirror reaction duration is 25 min (Supplementary Figure S11).

**FIGURE 3:**
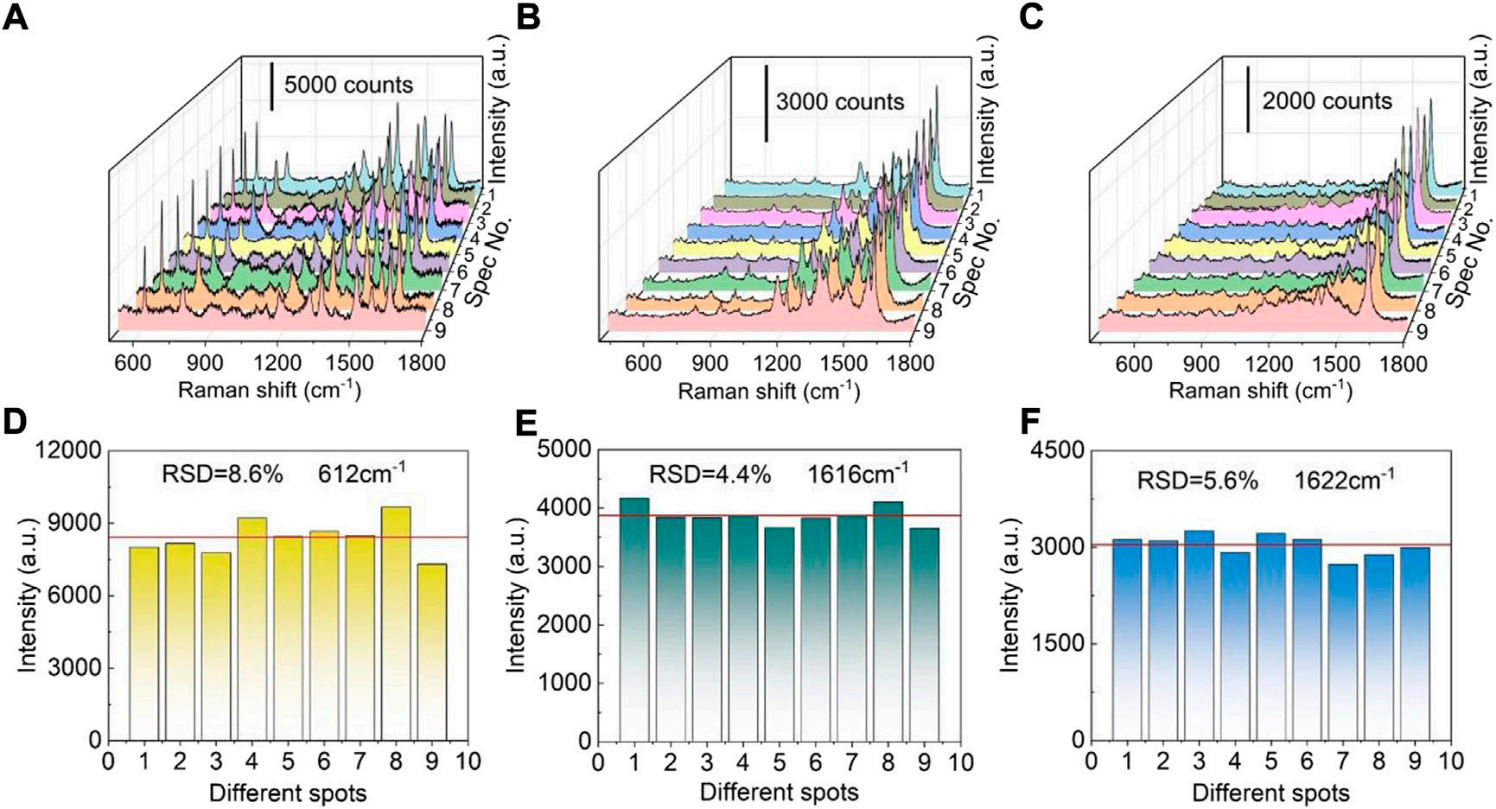
SERS signal uniformity of the Ag nanoparticles modified TiO_2_ nanotube arrays. **(A)** SERS spectra of R6G (10^−7^ M) at different positions. **(B)** SERS spectra of MG (10^−5^ M) at different positions. **(C)** SERS spectra of MB (10^−5^ M) at different positions. **(D)** The 612 cm^−1^ band intensities of R6G (10^−7^ M) recorded from different locations. **(E)** The 1,616 cm^−1^ band intensities of MG (10^−5^ M) recorded from different locations. **(F)** The 1,622 cm^−1^ band intensities of MB (10^−5^ M) recorded from different locations.

As an efficient SERS substrate, the uniformity of SERS signal is very important. For evaluating SERS signal uniformity, R6G was employed as a probe molecule. Figure 3A displays nine SERS spectra which were collected from nine randomly selected sites on the fabricated SERS substrate. The relatively strong Raman peak at 612 cm^−1^ can be attributed to in-plane and out-of-plane xanthene ring deformation (Thrall et al., 2012), which can be still distinguishable under ultralow concentrations. As shown in Figure 3D, the peak intensities at 612 cm^−1^ possess a low relative standard deviation (RSD) value of 8.6%. The peak intensities are almost uniformly distributed around the red horizontal line which represents the average intensity, directly indicating the fabricated SERS substrate has good SERS-signal uniformity. Moreover, malachite green (MG, 10^−5^ M) and methylene blue (MB, 10^−5^ M) are also used as probe molecules to further investigate the uniformity of the fabricated SERS substrates. For MG, the relatively strong Raman peak at 1616 cm^−1^ is assigned to the ring stretching of C-C (Sivashanmugan et al., 2015). Similarly, the peak at 1622 cm^−1^ of MB is similar to that of MG, probably also due to ring stretching of C-C (Xu et al., 2019). Statistical analysis was carried out for the peak intensities at 1616 cm^−1^ and 1,622 cm^−1^. RSD values of MG (1,616 cm^−1^) and MB (1,622 cm^−1^) were calculated to be 4.4 and 5.6% (Figures 3E,F), respectively, further confirming the good SERS-signal uniformity of the fabricated hybrid SERS substrate.

**FIGURE 4:**
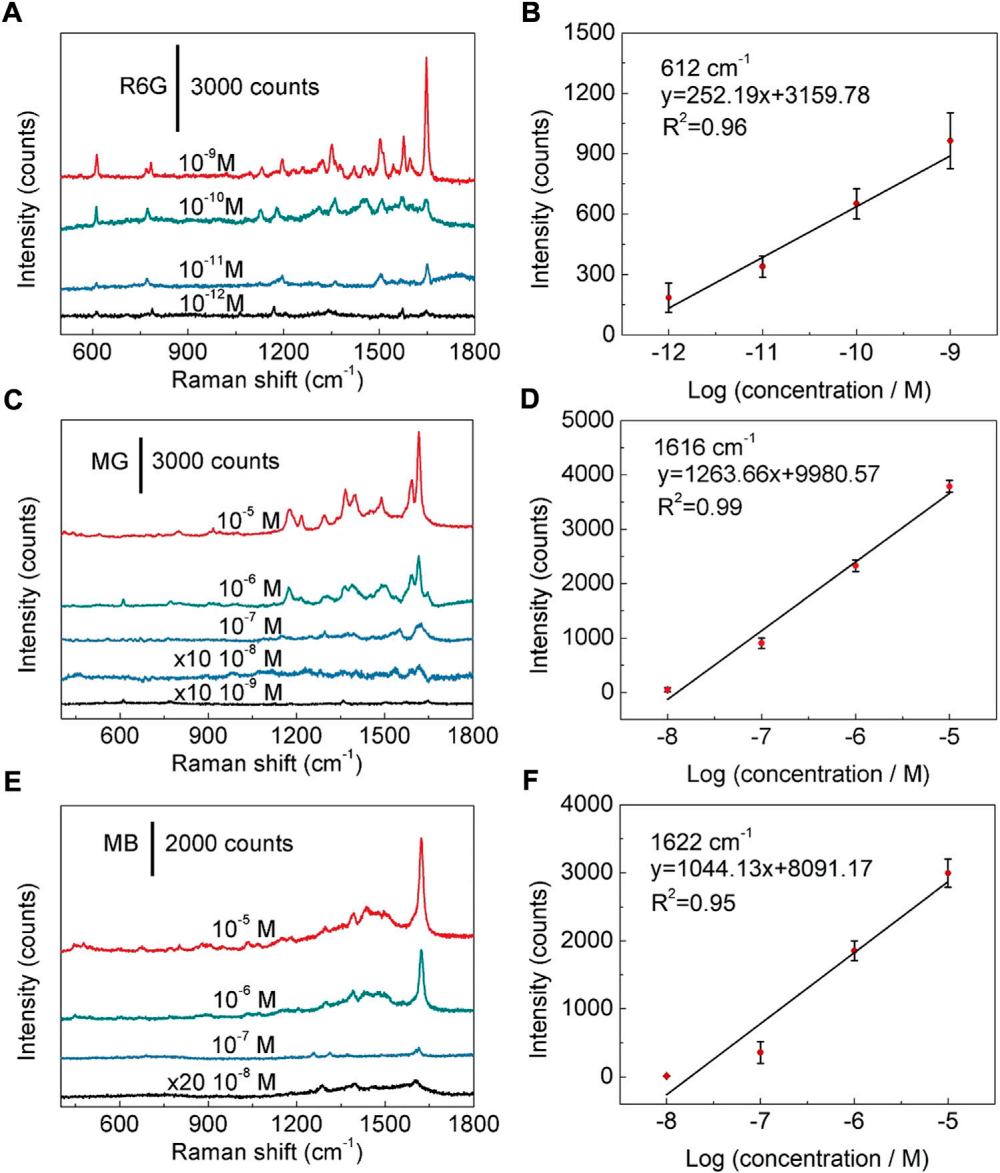
SERS sensitivity to organic dyes. **(A)** SERS spectra of R6G at concentrations of 10^−9^ M-10^−12^ M. **(B)** Linear relationship between the peak intensity and the logarithmic concentration of R6G. **(C)** SERS spectra of MG at different concentrations from 10^−5^ M to 10^−9^ M. **(D)** Linear relationship between the peak intensity and logarithmic concentration of MG. **(E)** SERS spectra of MB with the concentration ranging from 10^−5^ M to 10^−8^ M. **(F)** Linear relationship between the band intensity and logarithmic concentration of MB.

Next, SERS sensitivity of the fabricated SERS substrate was examined. The detectable concentration limit for R6G molecule is as low as 10^−12^ M (Figure 4A), revealing that the fabricated Ag nanoparticles/TiO_2_ nanotubes hybrid nanostructures possess high sensitivity to R6G. The relationship between spectral peak intensity and logarithmic concentration of R6G is roughly a straight line (*R* ^2^ = 0.96) (Figure 4B), which proves that the Ag nanoparticles modified TiO_2_ nanotube arrays have the ability of quantitative detection of R6G (Hildebrandt and Stockburger, 1984; Kumar et al., 2020b). The quantitative detection can also be realized for MG and MB. As shown in Figures 4C,E, detectable concentration limits for MG and MB are estimated to be 10^−9^ M and 10^−8^ M, respectively. The linear fitting results show that the *R* ^2^ values are 0.99 and 0.95 for MG and MB, respectively (Figures 4D,F and Supplementary Figure S12). Compared with the detectable concentration limits or LODs of R6G achieved using the other metal-doped TiO_2_ hybrids reported in the previous literatures, our Ag nanoparticles modified TiO_2_ nanotube array substrate has a comparable or superior detectable concentration limits (Supplementary Table S1). Furthermore, for exploring the Raman enhancement effect of Ag nanoparticles modified TiO_2_ nanotube arrays, R6G (1 × 10^−2^ M) was dropped on the polyvinyl chloride film, its Raman signal was detected and compared with (1 × 10^−10^ M) the SERS signal of R6G on the Ag nanoparticles modified TiO_2_ nanotube arrays. The result reveals that the substrate has an obvious enhancement effect, and its enhancement factor (EF) is estimated to be 1.4×10^8^ (Supplementary Figure S13A and Part 3: Estimation of enhancement factor in the Supporting Information). In order to further verify such result, EF was estimated by using R6G molecules excited by a 785 nm laser (Supplementary Figure S13B and Part 3: Estimation of enhancement factor in the Supporting Information) (Le Ru et al., 2007). The results show the EF calculated using the 785 nm excitation laser is 1.68×10^8^, which is comparable with that achieved using a 532 nm excitation laser. It is found that the enhancement effect mainly comes from Ag nanoparticles (Supplementary Figures S14, S15), which further indicates that high-density Ag nanoparticles can produce high-density hot spots, and thus can significantly enhance Raman signal of analyte molecules and improve detection sensitivity.

**FIGURE 5:**
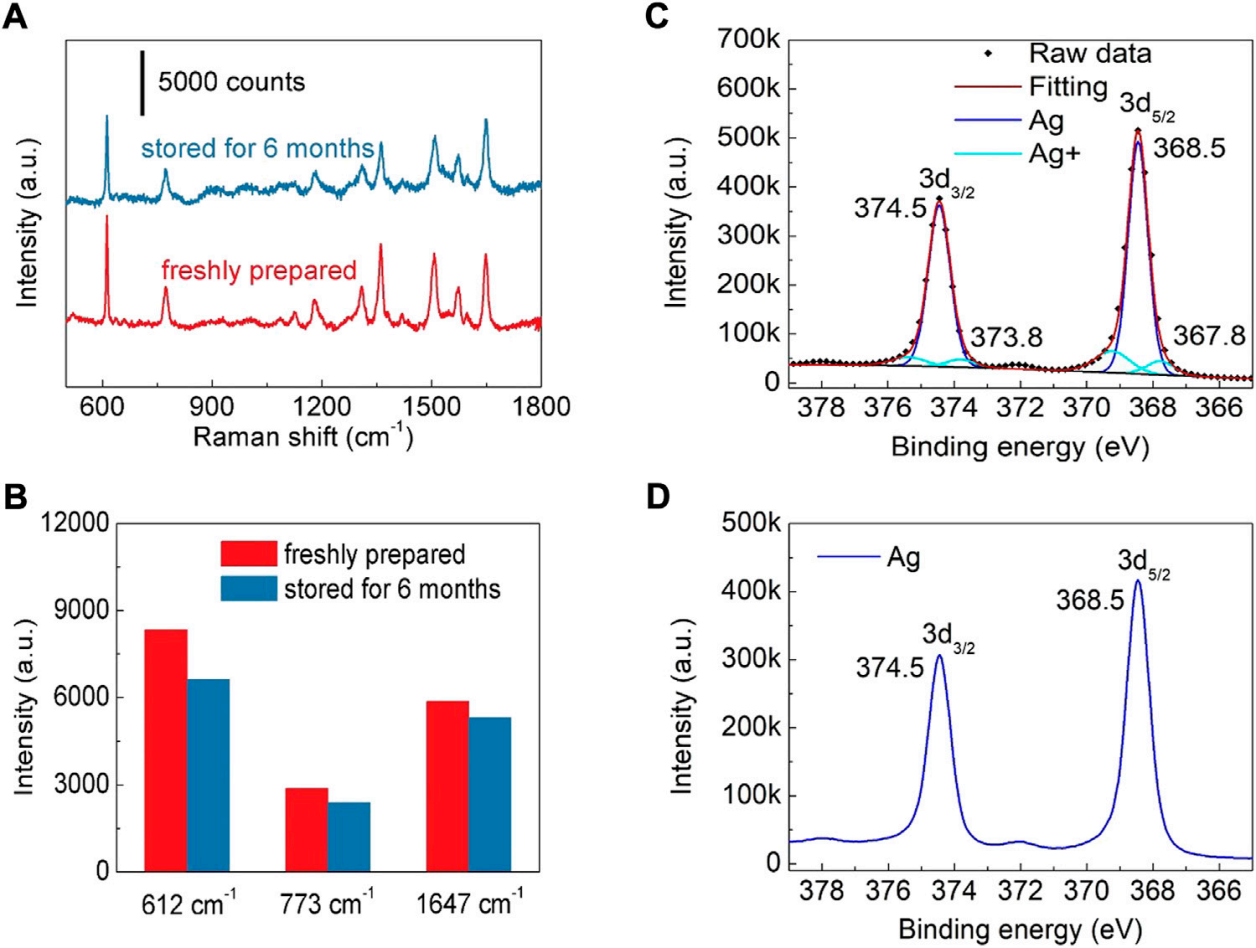
**(A)** SERS spectra of R6G (10^−7^ M) collected from the freshly prepared substrate and the one kept for 6 months **(B)** The intensity of R6G peaks at 612, 773 and 1,647 cm^−1^. **(C)** The Ag 3days XPS spectrum of the substrate stored for 6 months **(D)** The Ag 3days XPS spectrum of the freshly prepared substrate.

In addition to the sensitivity of a SERS substrate, storage stability is another important factor to evaluate its potential for practical application. The SERS spectra of R6G (10^−7^ M) collected from the freshly prepared substrate and the one kept for 6 months are compared in Figure 5A. It can be found that after being stored for 6 months, Ag nanoparticles modified TiO_2_ nanotube array substrate still maintain high SERS activity (Figure 5A). Comparing with the freshly prepared substrate, the intensities of 612, 773 and 1,647 cm^−1^ do not show obvious reduction (Figure 5B), indicating that Ag nanoparticles modified TiO_2_ nanotube arrays have good storage stability. The decrease of SERS activity is attributed to the oxidation of Ag nanoparticles. As reported in previous studies, the oxidation of Ag nanostructure has moderately reduced SERS activity (Erol et al., 2009; Han et al., 2011). In order to confirm the oxidation of the Ag nanoparticles modified on TiO_2_ nanotube, the freshly prepared sample and the one stored for 6 months are characterized by a X-ray photoelectron spectrometer (XPS) (Figures 5C,D). It can be found that after 6 months of storage, only a tiny percentage of Ag atoms on the surface of Ag nanoparticles are oxidized, indicating that the fabricated Ag nanoparticles modified TiO_2_ nanotube arrays have good storage stability. Moreover, the fabricated SERS substrate also has good chemical stability in both acidic and alkaline solutions. After soaking for 6 h in a KOH (pH = 13) or a H_3_BO_3_ (pH = 1) solution, the morphology of Ag nanoparticles modified on the TiO_2_ nanotubes was not changed obviously (Supplementary Figure S16), confirming that the fabricated SERS substrate has excellent acid and alkali resistance. Meanwhile, the SERS activity is also well maintained after acid or alkali solution treatment (Supplementary Figure S17), indicating that the as-prepared SERS substrate can be employed in a wide range of environments.

**FIGURE 6:**
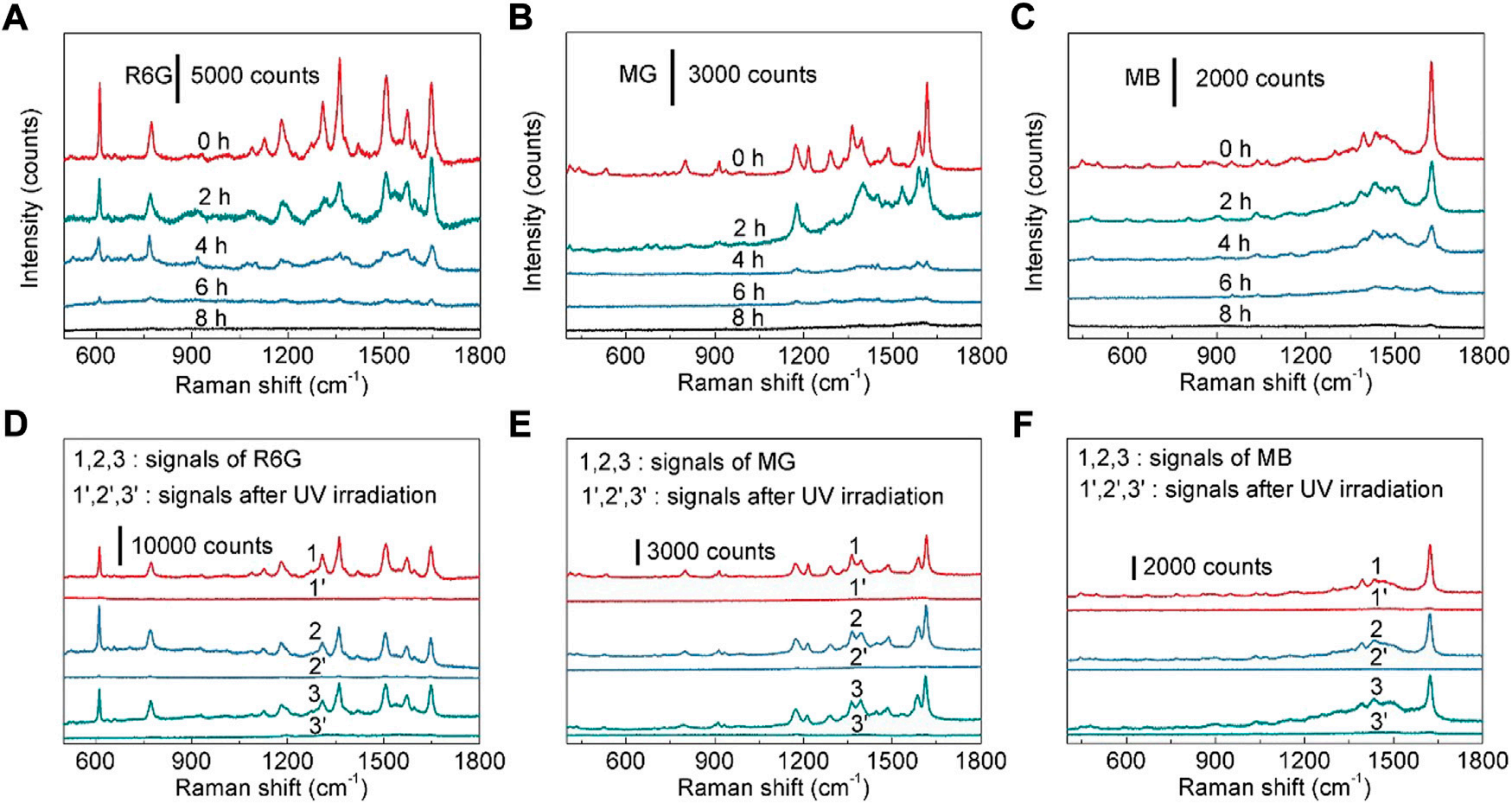
**(A)** SERS spectra of R6G (10^−7^ M) collected from the as-prepared Ag nanoparticles modified TiO_2_ nanotube arrays after UV irradiation for 0–8 h **(B)** SERS spectra of MG (10^−5^ M) collected from the as-prepared Ag nanoparticles modified TiO_2_ nanotube arrays after UV irradiation for 0–8 h **(C)** SERS spectra of MB (10^−5^ M) collected from the as-prepared Ag nanoparticles modified TiO_2_ nanotube arrays after UV irradiation for 0–8 h **(D)** SERS spectra of R6G (10^−7^ M) during three self-cleaning cycles. **(E)** SERS spectra of MG (10^−5^ M) during three self-cleaning cycles. **(F)** SERS spectra of MB (10^−5^ M) during three self-cleaning cycles.

The self-cleaning performance is also an important index to a reusable SERS substrate (Ouyang et al., 2017; Sun et al., 2017; Han et al., 2018). The photocatalytic property of TiO_2_ nanotubes enables the ability for the photocatalytic degradation of organic molecules. As shown in Figure 6A, with the increase of UV irradiation (220 mW cm^−2^) time, the Raman signal intensity of R6G decreases accordingly, indicating that the R6G molecules absorbed on the SERS substrate could be degraded by UV irradiation. After UV lamp irradiation for 8 h, no Raman signal of R6G can be detected (Figure 6A), reflecting that the Ag nanoparticles modified TiO_2_ nanotube arrays can be used as an effective reusable SERS substrate. The photocatalytic degradation of R6G molecule by Ag nanoparticles modified TiO_2_ nanotube arrays can be explained by that the light excitation of “hot electrons” from Ag nanoparticles to TiO_2_ is captured by oxygen to form hydroxyl radicals (Pan et al., 2012). These hydroxyl radicals in principle are highly effective for organic species decomposition downwards to simple molecules, such as H_2_O and CO_2_ (Zheng et al., 2007; Kochuveedu et al., 2012; Pan et al., 2012; Kumar et al., 2016; Shvalya et al., 2020). At the same time, the recyclability of substrate is also investigated. By repeating the cycle of loading R6G (10^−7^ M) on a SERS substrate and photocatalytic degradation, three self-cleaning cycles were performed on the same SERS substrate. The experimental result confirms that the SERS activity of the reused SERS substrate is very close to that of the fresh SERS substrate (Figure 6D). Then the influence of UV irradiation to the morphology of Ag nanoparticles modified TiO_2_ nanotube arrays was also studied. It is found that there is no obvious change in the morphologies of Ag nanoparticles after UV irradiation (Supplementary Figure S18). Therefore, the UV lamp irradiation for three times will not remarkably change the micro-structure of the fabricated Ag nanoparticles modified TiO_2_ nanotube arrays. When the photodegradation recycle was repeated for more than 3 times, the SERS activity of the reused substrate decreased (the peak intensity of R6G was reduced to ∼92% of that form the fresh substrate). Meanwhile, MG and MB were also used to evaluate the reusability of the fabricated SERS substrate. The SERS signals of MG (10^−5^ M) and MB (10^−5^ M) are lost after the UV lamp irradiation for 8 h, and the SERS activities of the SERS substrate after first, second and third self-cleaning are very similar (Figures 6B,C,E,F). Therefore, Ag nanoparticles modified TiO_2_ nanotube arrays can be employed as recyclable SERS substrates, which is beneficial to reducing the cost of SERS substrate. In order to shorten the UV irradiation duration, after being soaked in an aqueous solution of MG (10^−5^ M) for 3 h, the SERS substrate was immersed in deionized water and irradiated with UV lamp at room temperature for a certain time to remove the absorbed molecules on the SERS substrate. It is found that after UV irradiation for 2.5 h, the SERS signal of MG is disappeared (Supplementary Figure S19). Compared with the previous results (Figure 6E), the photocatalytic degradation efficiency of TiO_2_ nanotubes in deionized water is much higher. Besides, the photocatalytic degradation efficiency of our SERS substrate is comparable to or higher than those reported in the previous literatures (Supplementary Table S2).

## Conclusion

In conclusion, a large-area SERS substrate composed of Ag nanoparticles modified TiO_2_ nanotube arrays was fabricated by sacrificial ZnO nanocone template-assisted TiO_2_ deposition. The prepared Ag nanoparticles/TiO_2_ nanotubes hybrid nanostructures have high SERS sensitivity and good spectral uniformity. The fabricated SERS substrate showed good linear response between the characteristic peak intensity and the logarithmic concentration of dyes (rhodamine 6G, malachite green and methylene blue). The analyte molecules such as malachite green adsorbed on the Ag nanoparticles modified TiO_2_ nanotube arrays can be photocatalytic degradation after UV irradiation for 2.5 h in deionized water, demonstrating that the prepared hybrid nanostructures can serve as recyclable SERS substrates. The fabricated Ag nanoparticles modified TiO_2_ nanotube arrays also have good acid and alkali resistance and thus can be employed to detect molecules in a wide range of environments. The fabricated Ag nanoparticles modified TiO_2_ nanotube arrays have potential applications in environmental monitoring, analytical chemistry and other fields. There are also some limitations which need to be investigated in the future research. Firstly, as a reusable SERS substrate, it is necessary to further reduce the self-cleaning duration and increase the number of self-cleaning cycles. Additionally, as an important factor to evaluate the practical application ability of the fabricated SERS substrate, the detectable concentration limit is still needed to be further lowered.

## Data Availability

The original contributions presented in the study are included in the article/Supplementary Material, further inquiries can be directed to the corresponding authors.
