# Supplementary material for: Arrays of Ag-nanoparticles decorated TiO2 nanotubes as reusable three-dimensional surface-enhanced Raman scattering substrates for molecule detection
Source: Front Chem. 2022 Oct 3;10:992236. doi: 10.3389/fchem.2022.992236 (PMC9574249; doi:10.3389/fchem.2022.992236)
Supplement: Supplementary file 1 [file Table1.docx]

Supporting information

**Arrays of Ag-nanoparticles decorated TiO_2_ nanotubes as reusable three-dimensional surface-enhanced Raman scattering substrates for molecule detection**

Haichao Zhai,^a^ Chuhong Zhu,^a,^* Xiujuan Wang,^b,^* Yupeng Yuan,^a^ Haibin Tang ^c,^*

^a^College of Chemistry & Chemical Engineering, and School of Materials Science and Engineering, Anhui University, Hefei, Anhui 230601, China

^b^School of Microelectronics, Hefei University of Technology, Hefei 230601, China

^c^Key Laboratory of Materials Physics, and Anhui Key Laboratory of Nanomaterials and Nanotechnology, Institute of Solid State Physics, HFIPS, Chinese Academy of Sciences, P. O. Box 1129, Hefei 230031, People’s Republic of China

*Corresponding authors. E-mail addresses: chzhu@ahu.edu.cn (Chuhong Zhu); hbtang@issp.ac.cn (Haibin Tang); xjwang@issp.ac.cn (Xiujuan Wang).

The Supporting Information includes:

Part 1: Figure S1-S19

Part 2: Table S1-S2

Part 3: Estimation of enhancement factor

Part 4: References

**Part 1**


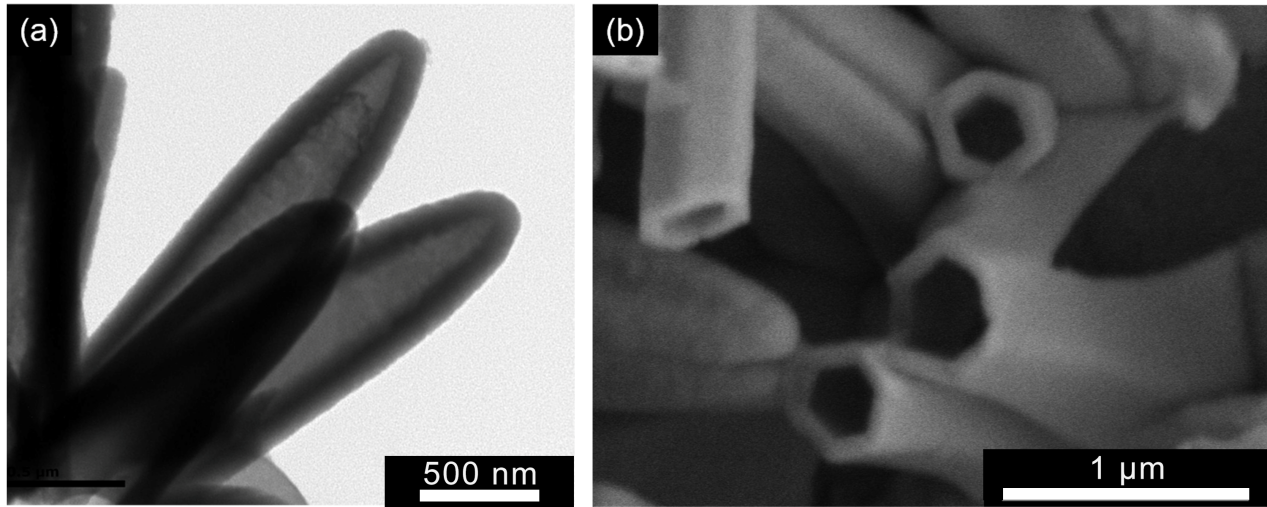


**Figure S1.** (a) TEM image of single TiO_2_ nanotube; (b) SEM image of broken TiO_2_ nanotubes.

**
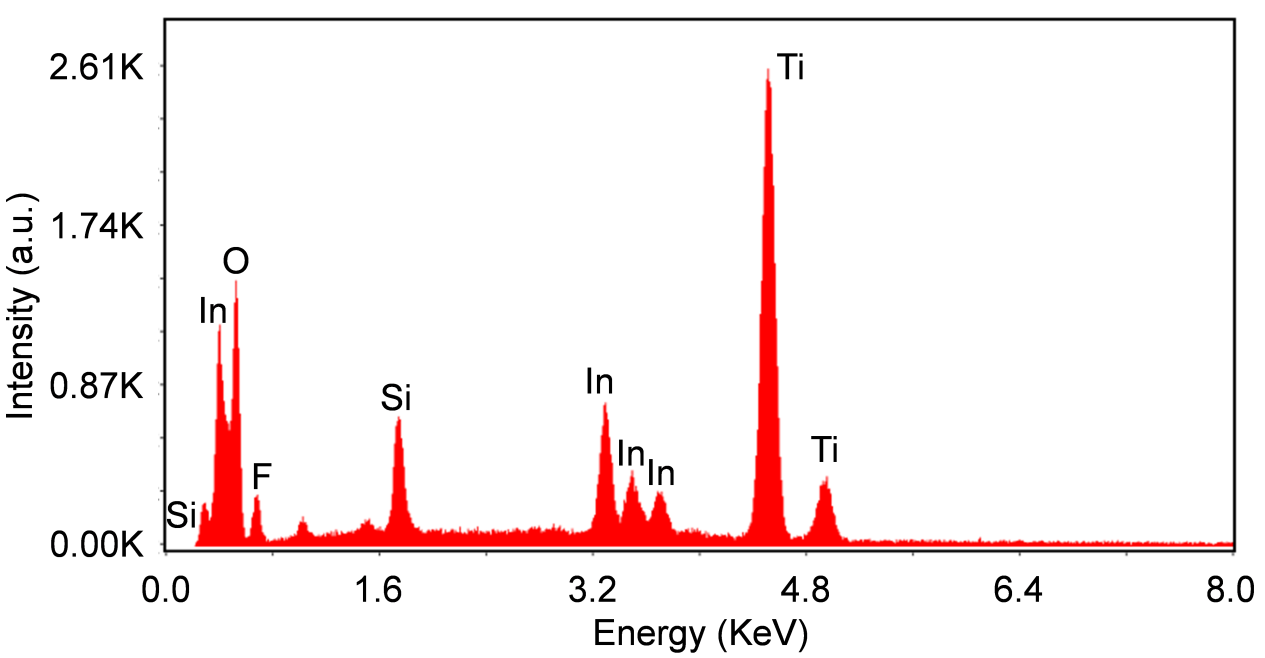
Figure S2.** The energy dispersive spectrometry (EDS) spectrum of the fabricated TiO_2_ nanotube arrays on an ITO glass.


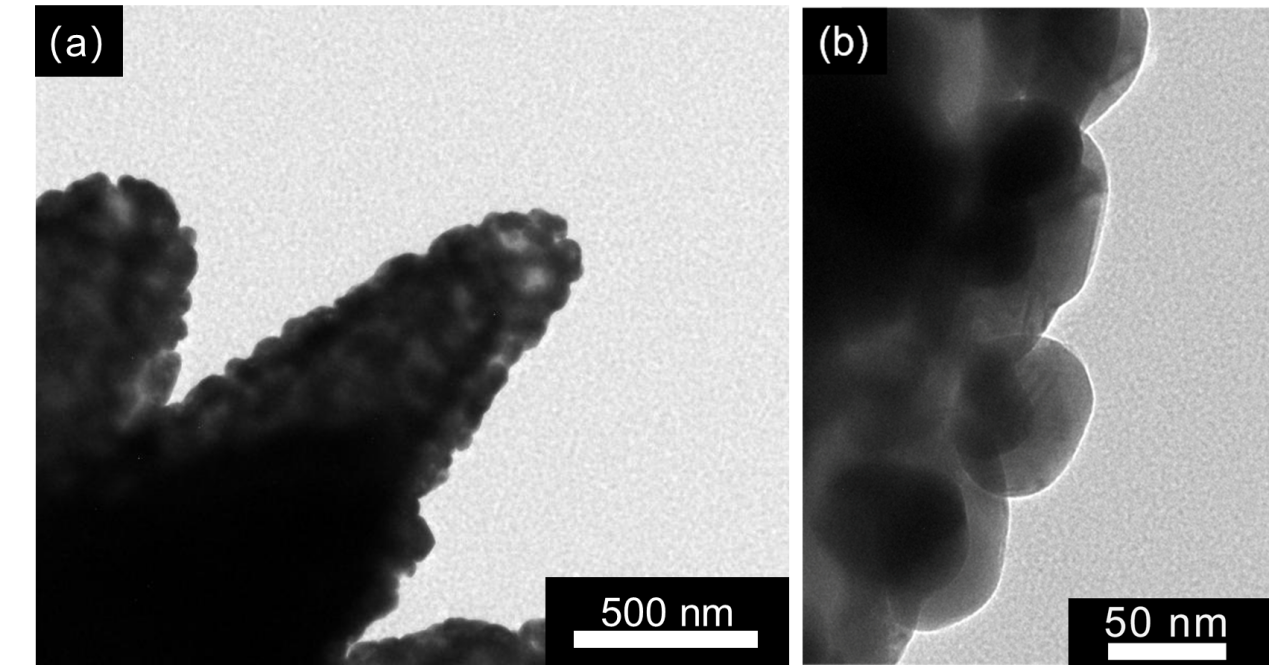


**Figure S3.** (a, b) TEM images of Ag nanoparticles modified TiO_2_ nanotubes.


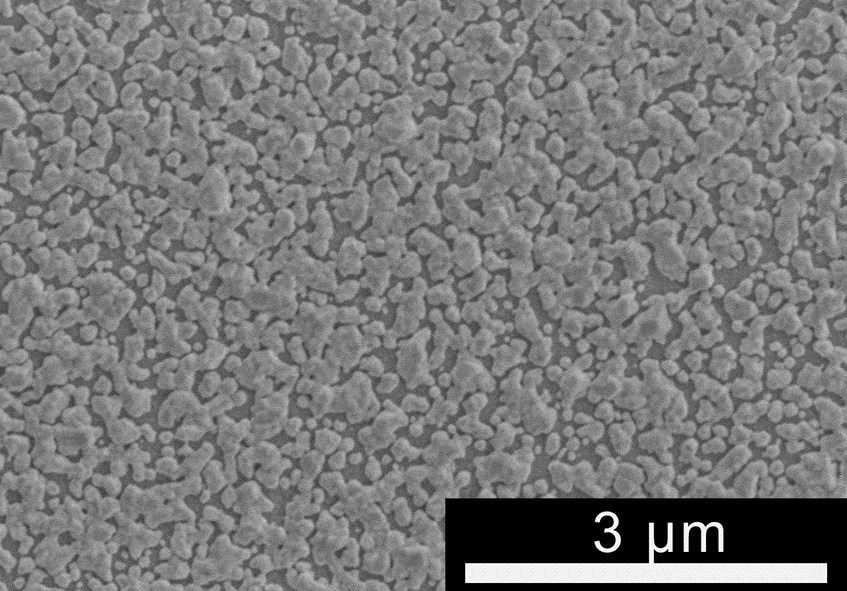


**Figure S4.** SEM image of Ag nanoparticles grown on a flat ITO substrate with silver nitrate concentration of 20 mmol/L, under 50 ^o^C and with silver mirror reaction of 25 minute.


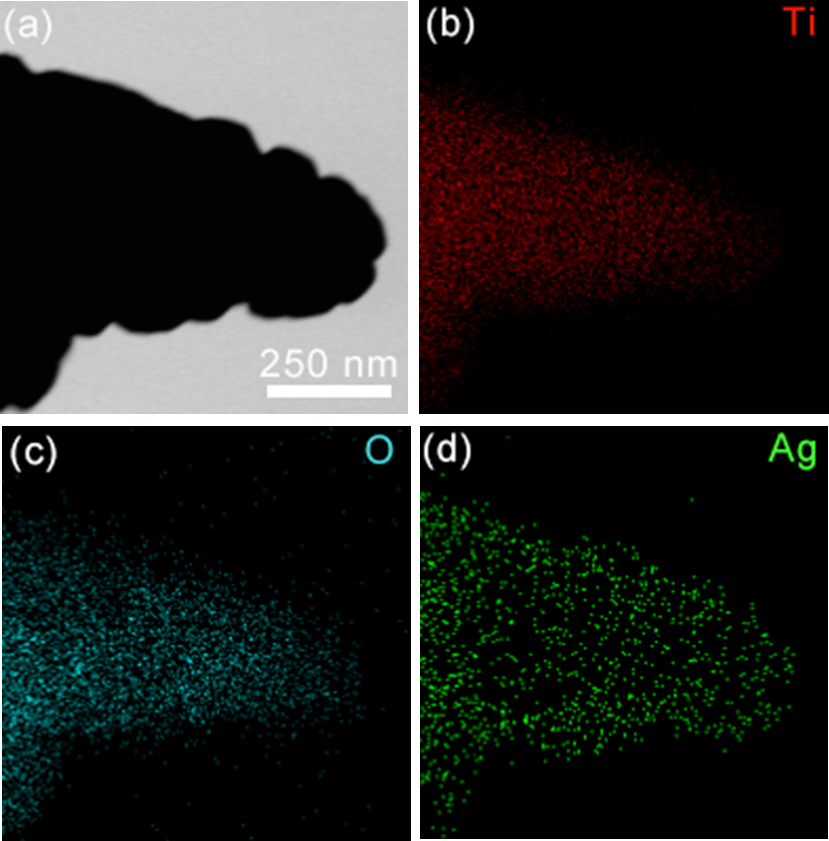


**Figure S5.** Energy dispersive spectrometry (EDS) mapping images of Ag nanoparticles modified TiO_2_ nanotube arrays: (a) bright-field image; (b) Ti element mapping; (c) O element mapping; (d) Ag element mapping.


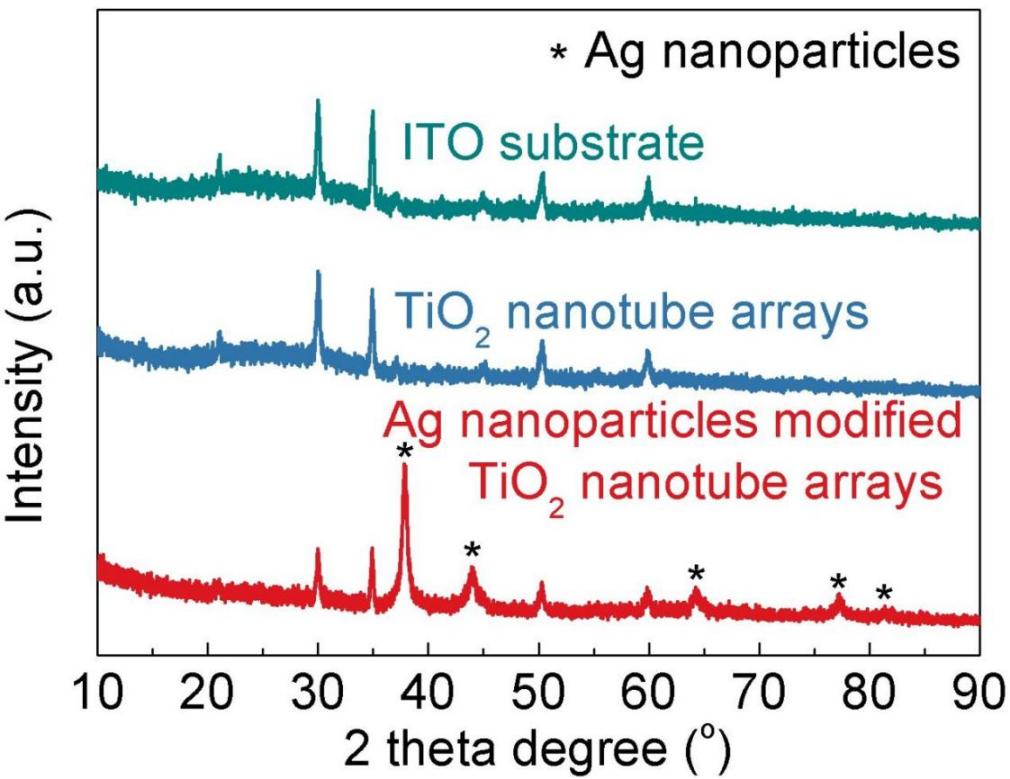


**Figure S6.** XRD spectra of an ITO substrate, TiO_2_ nanotube arrays and Ag nanoparticles modified TiO_2_ nanotube arrays.


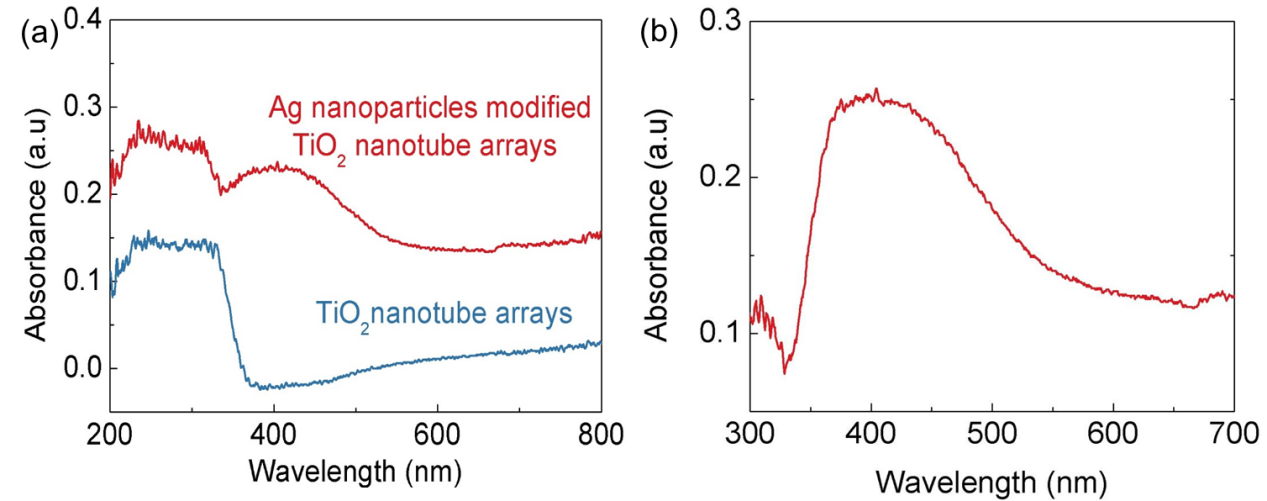


**Figure S7.** Optical absorption spectra of: (a) the TiO_2_ nanotube arrays and the Ag nanoparticles modified TiO_2_ nanotube arrays; (b) the Ag nanoparticles (obtaining by subtracting the bottom curve from the top curve shown in (a)).


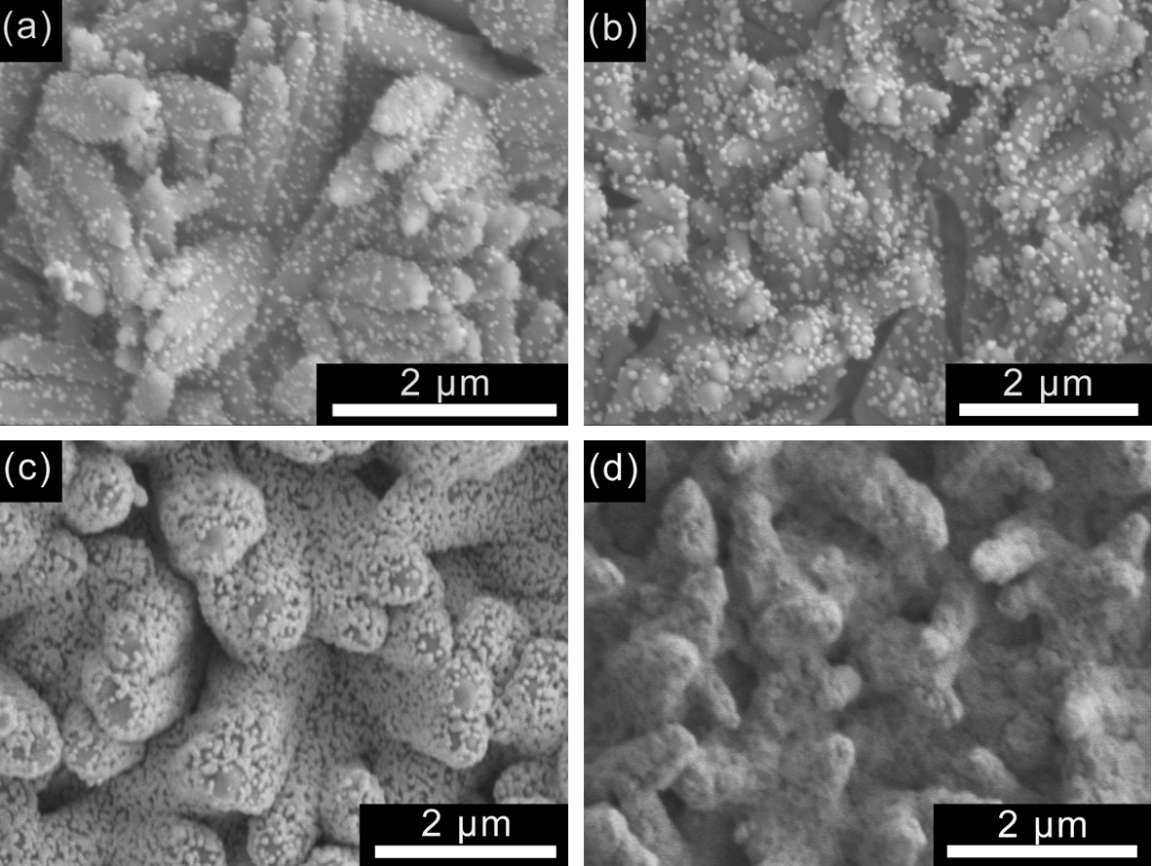


**Figure S8.** SEM images of Ag nanoparticles modified TiO_2_ nanotube arrays prepared under the same silver mirror reaction duration (25 min), while with different concentrations of AgNO_3_: (a) 5 mmol/L; (b) 10 mmol/L; (c) 20 mmol/L; (d) 50 mmol/L.


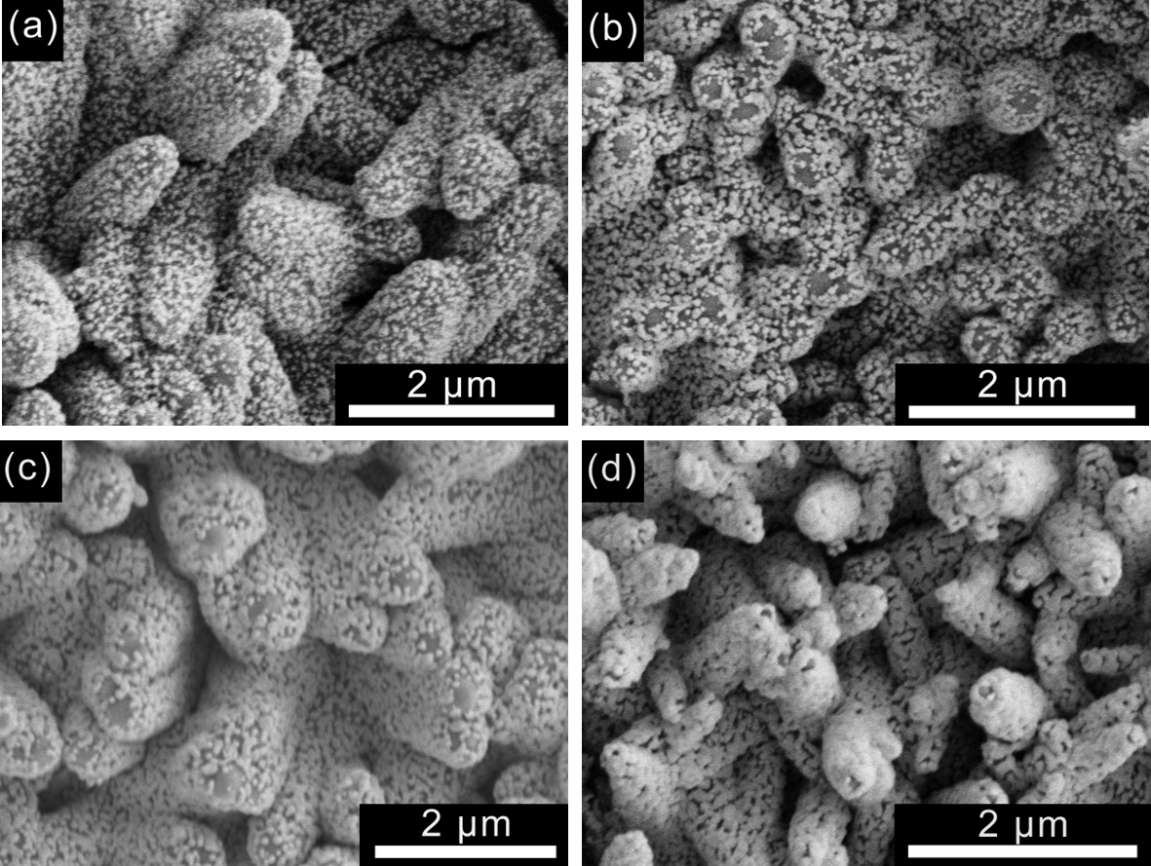


**Figure S9.** SEM images of Ag nanoparticles modified TiO_2_ nanotube arrays prepared under the same concentration of AgNO_3_ (20 mmol/L), while with different silver mirror reaction durations: (a) 5 min; (b) 10 min; (c) 25 min; (d) 50 min.


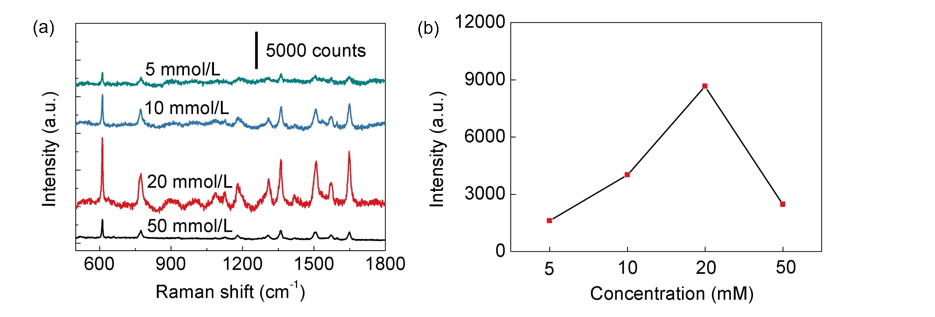
 **Figure S10.** SERS spectra of R6G (10^-7^ M) collected from Ag nanoparticles modified TiO_2_ nanotube arrays prepared under the same silver mirror reaction duration (25 min), while with different concentrations of AgNO_3_; (b) the 612 cm^-1^ peak intensity of R6G (10^-7^ M).


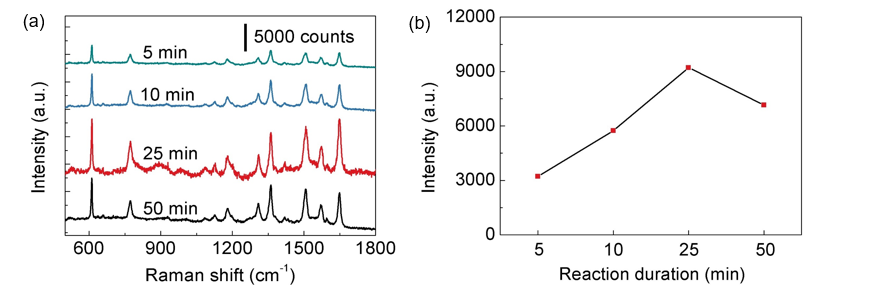
 **Figure S11.** (a) SERS spectra of R6G (10^-7^ M) collected from Ag nanoparticles modified TiO_2_ nanotube arrays prepared under the same concentration of AgNO_3_ (20 mmol/L), while with different silver mirror reaction durations; (b) the 612 cm^-1^ peak intensity of R6G (10^-7^ M).


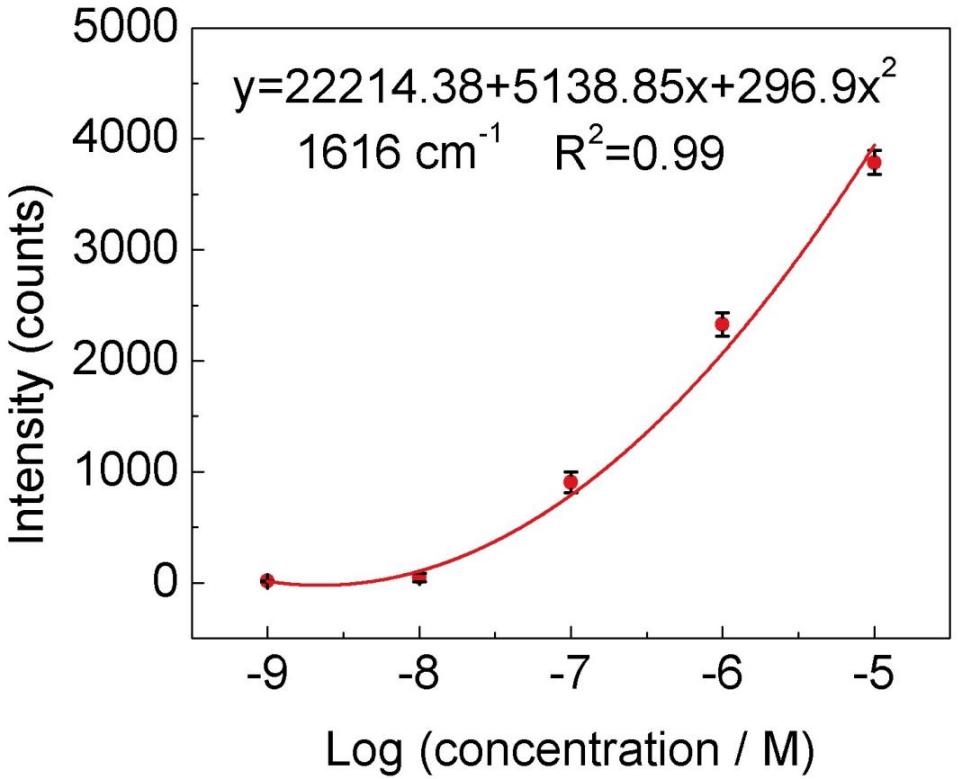


**Figure S12.** Relationship between MG concentration and logarithmic intensity of 1616 cm^-1^ characteristic peak.


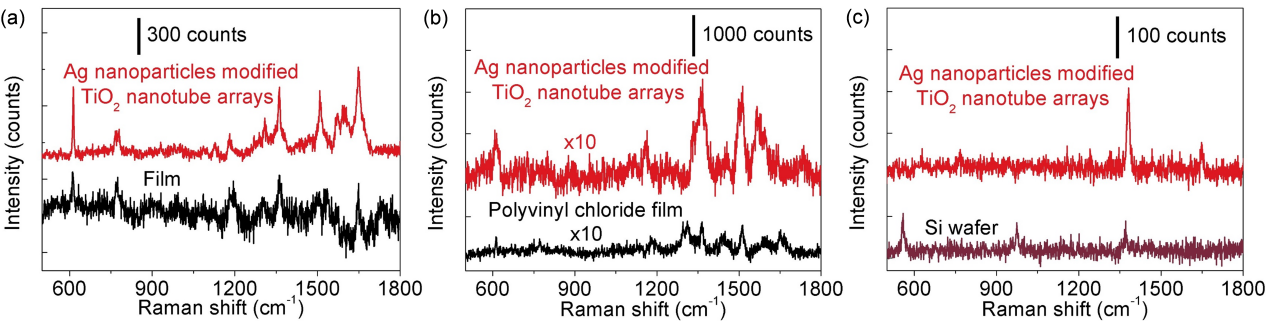


**Figure S13.** (a) Normal Raman spectrum of R6G on the polyvinyl chloride film and SERS spectrum of R6G adsorbed on the Ag nanoparticles modified TiO_2_ nanotube arrays achieved using a 532 nm excitation laser. (b) Normal Raman spectrum of R6G on the polyvinyl chloride film and SERS spectrum of R6G adsorbed on the Ag nanoparticles modified TiO_2_ nanotube arrays excited with a 785 nm excitation laser.


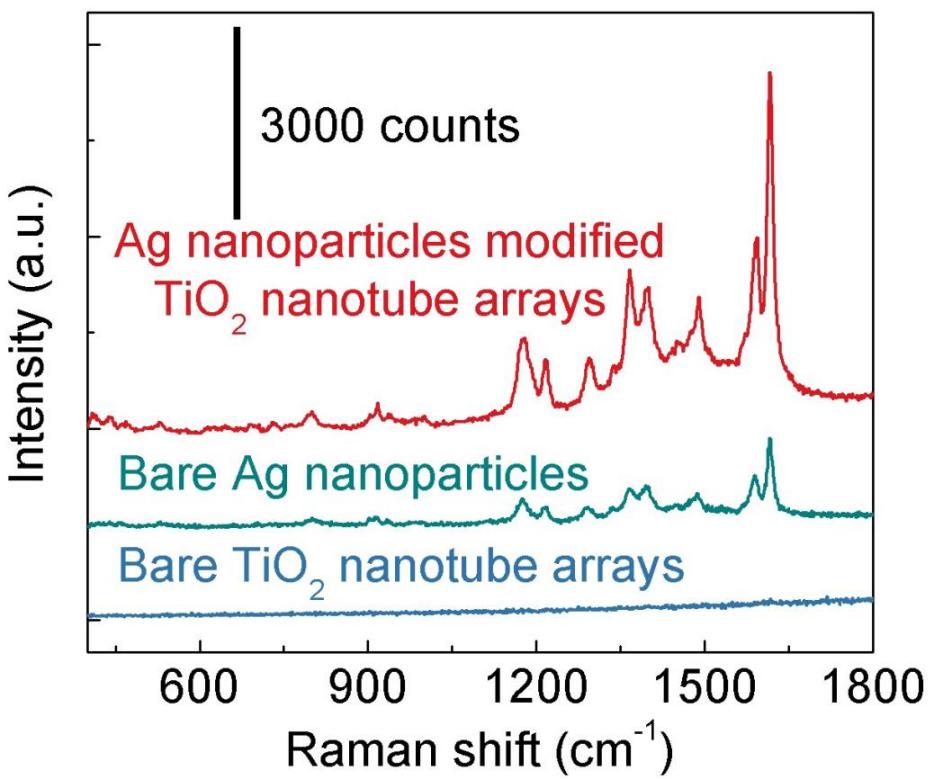


**Figure S14.** SERS spectra of MG (10^-5^ M) collected on the Ag nanoparticles modified TiO_2_ nanotube arrays, bare Ag nanoparticles and bare TiO_2_ nanotube arrays, respectively. The bare Ag nanoparticles grow on an ITO substrate were achieved under silver nitrate concentration of 20 mmol/L and silver mirror reaction condition of 50℃ for 25 minute.


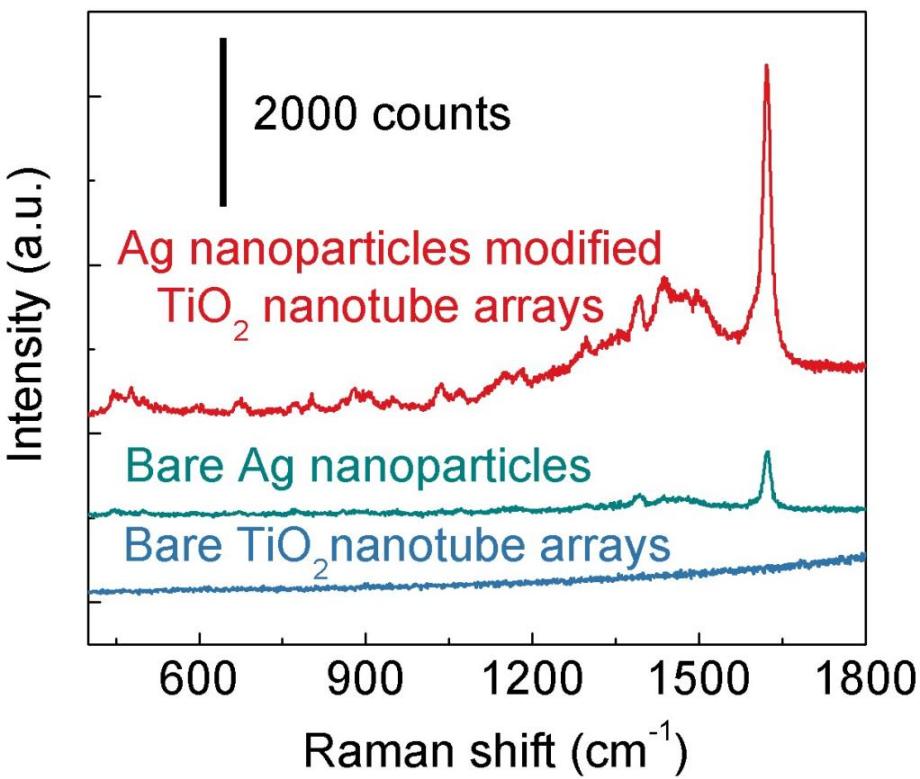


**Figure S15.** SERS spectrum of MB (10^-5^ M) collected on the Ag nanoparticles modified TiO_2_ nanotube arrays, bare Ag nanoparticles and bare TiO_2_ nanotube arrays, respectively. The bare Ag nanoparticles grow on an ITO substrate were prepared under silver nitrate concentration of 20 mmol/L and silver mirror reaction condition of 50℃ for 25 minute.


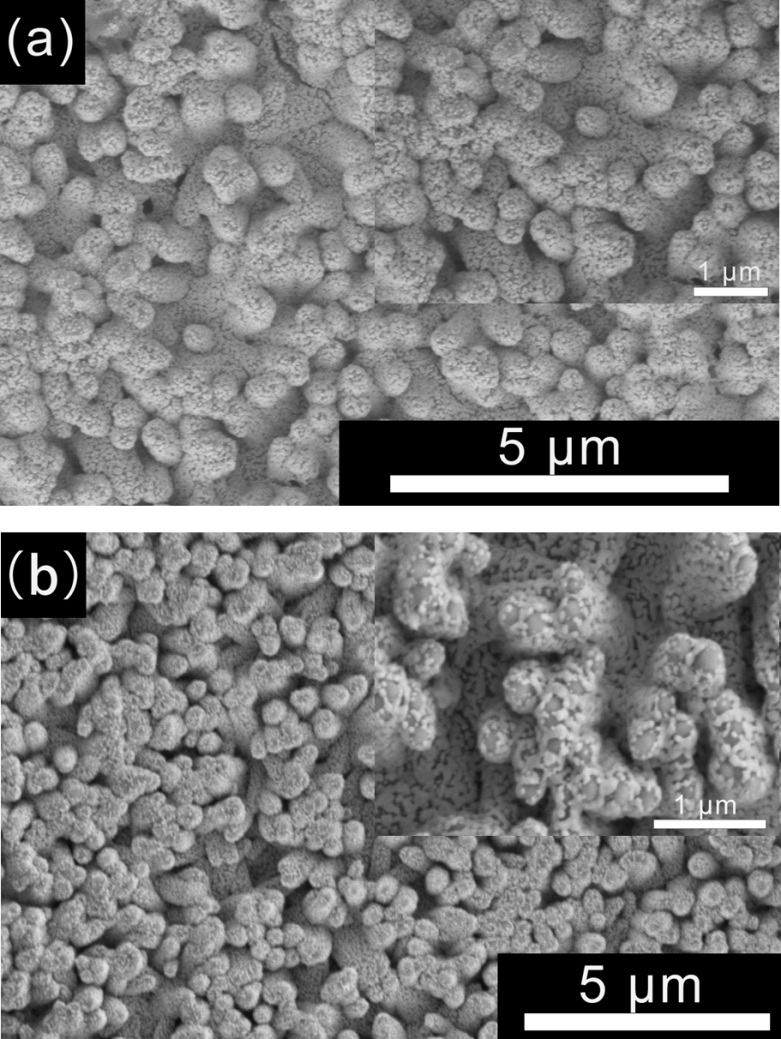


**Figure S16.** SEM images of Ag nanoparticles modified TiO_2_ nanotube arrays after being soaked for 6 hours in (a) a KOH solution (pH = 13) and (b) a H_3_BO_3_ solution (pH = 1).


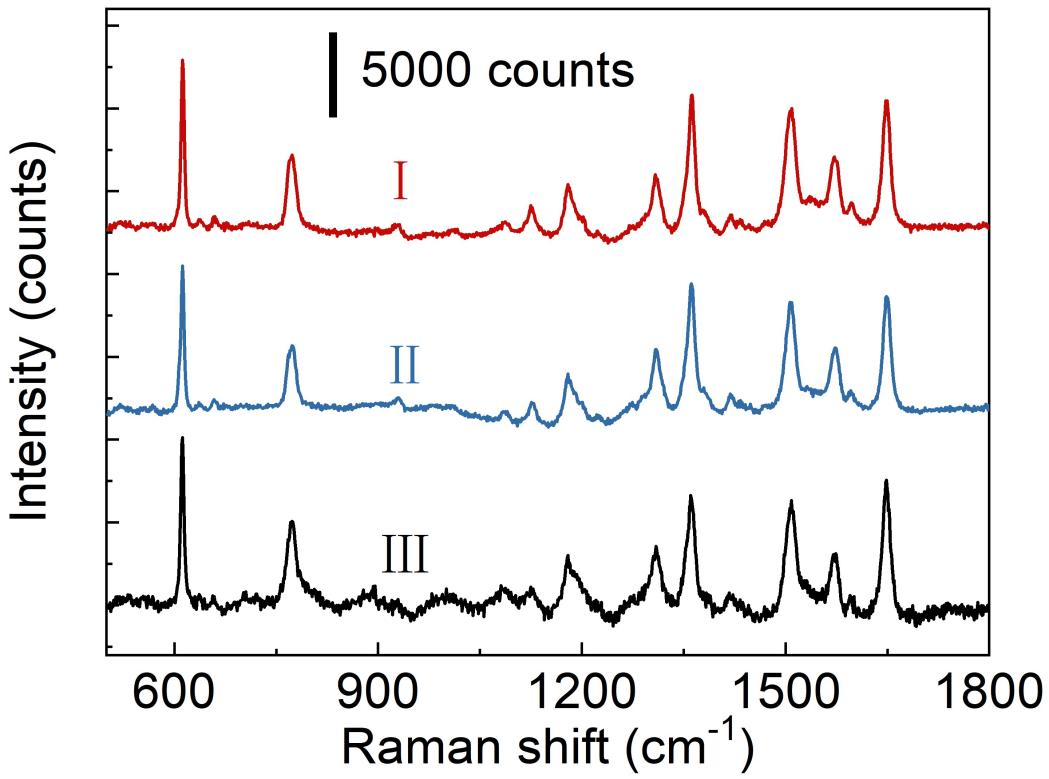


**Figure S17.** SERS spectra of R6G (10^-7^ M) collected from Ag nanoparticles modified TiO_2_ nanotube arrays after being soaked for 6 hours in (Ⅰ) a H_3_BO_3_ solution (pH = 1), (Ⅱ) a KOH solution (pH = 13), and (Ⅲ) from the SERS substrate without soaking.


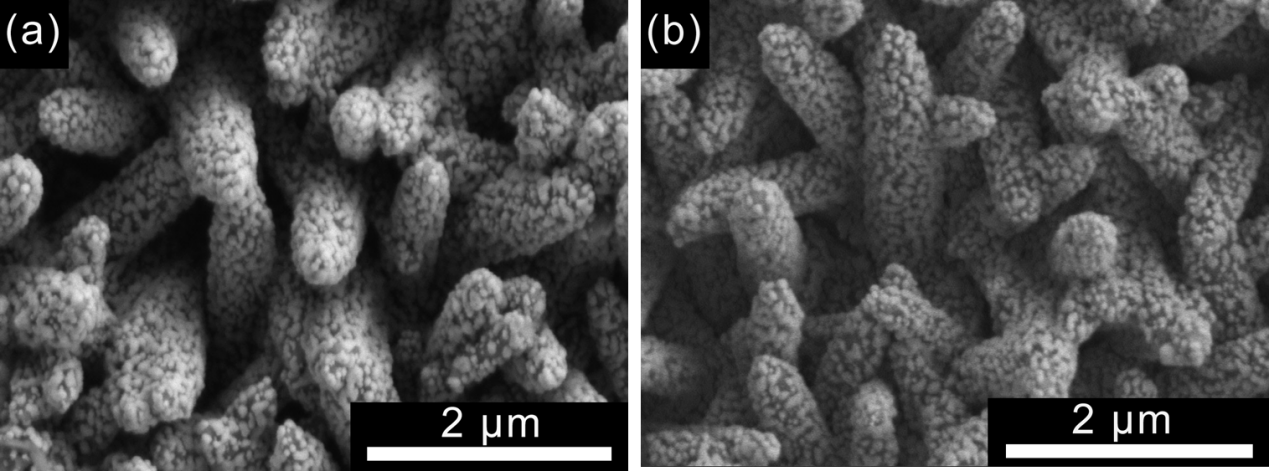


**Figure S18.** SEM images of the same Ag nanoparticles modified TiO_2_ nanotube arrays (a) before and (b) after UV irradiation for three times.


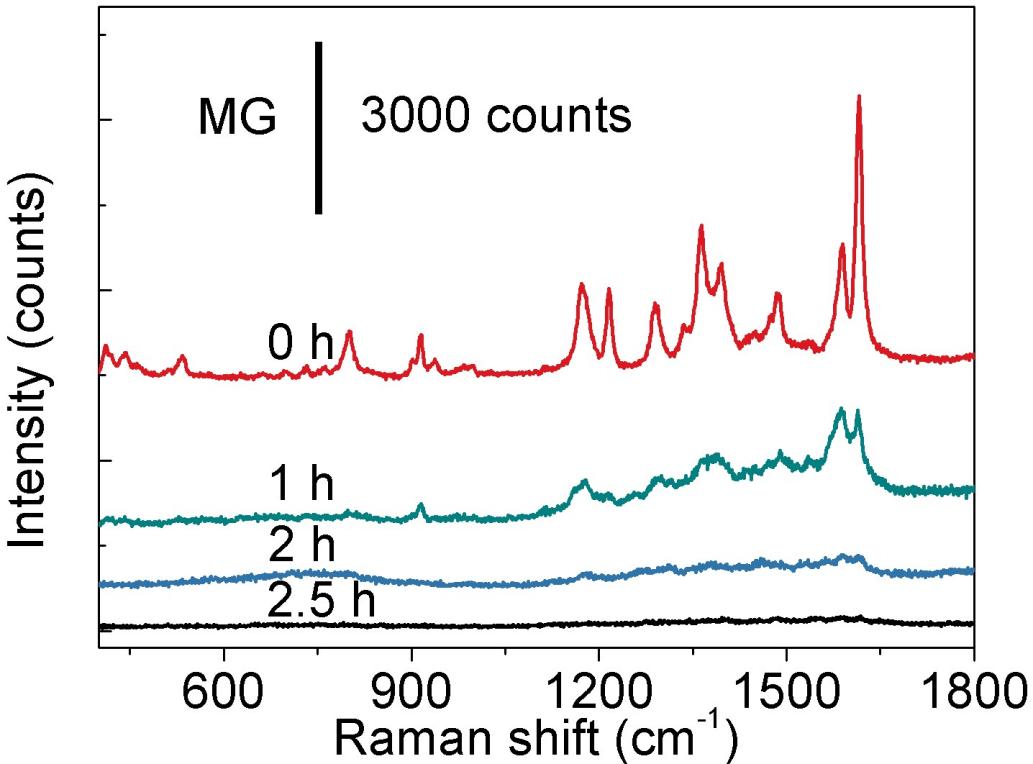


**Figure S19.** SERS spectra of MG (10^-5^ M) collected from the as-prepared Ag nanoparticles modified TiO_2_ nanotube arrays after UV irradiation for 0 - 2.5 h in deionized water.

**Part 2**

**Table S1.** Comparison of LODs or detectable concentration limits between various SERS substrates.

| SERS substrate | LOD or detectable concentration limit (R6G) | Ref. |
| --- | --- | --- |
| Ag nanoparticles modified TiO_2_ nanotube arrays | 10^-12^ M | This work |
| Pt@TiO_2_ NTA | 10^-9^ M | J. Cai et al. [1] |
| Au@TiO_2_ NRAs | 10^-9^ M | Z. Xie et al.[2] |
| TiO_2_/MoOx nano-heterojunctions | 10^-8^ M | S. Xie et al.[3] |
| Au@Ag/TiO_2_ NTs | 10^-10^ M | C. Wang et al.[4] |
| Pt/VA-MoS_2_/NTAs | 10^-9^ M | J. Dong et al.[5] |
| TiO_2_-Ag-GO substrate | 10^-8^ M | M. Zhang et al.[6] |
| TiO_2_/Au nanowire arrays | 10^-9^ M | X. Zhao et al.[7] |
| Three-dimensional Ag/TiO_2_ arrays | 10^-7^M | H. Fang et al.[8] |

**Table S2.** Comparison of degradation performance between various SERS substrates.

| SERS substrate | Degradation time (analyte molecule) | Ref. |
| --- | --- | --- |
| Ag nanoparticles modified TiO_2_ nanotube arrays | 150 min (MG) | This work |
| TiO_2_ /ZnO | 180 min (MG) | P. Bansal et al.[9] |
| TiO_2_-AgNPs-GO nanostructure | 180 min (CV) | M. Zhang et al.[6] |
| TiO_2_ nanorod films | 240 min (R6G) | J. Li et al.[10] |
| Au-Coated ZnO Nanorods | 180 min (MB) | G. Sinha et al.[11] |
| TiO_2_ NFSF/Ti@Ag NPs | 120 min (MG) | L. Jiang al et al.[12] |
| TiO_2_/Ag | 300 min (R6G) | Y. Zhao et al.[13] |

**Part 3**

**Estimation of enhancement factor**

In Figure S13(a), the enhancement factor (EF) of the Ag nanoparticles modified TiO_2_ nanotube arrays was determined by computing the ratio of SERS to normal Raman scattering (NRS) of R6G using the following expression:

$EF=\frac{I_{\mathrm{SERS}}/N_{\mathrm{SERS}}}{I_{\mathrm{NRS}}/N_{\mathrm{NRS}}}=\frac{I_{\mathrm{SERS}}}{I_{\mathrm{NRS}}}\times\frac{S_{\mathrm{SERS}}V_{\mathrm{NRS}}C_{\mathrm{NRS}}}{S_{\mathrm{NRS}}V_{\mathrm{SERS}}C_{\mathrm{SERS}}}$ (1)

where *I*_SERS_ and *I*_NRS_ correspond to the SERS and NRS intensities, respectively, normalized for acquisition time and laser power. For the SERS enhancement factor, 5 μL 10^-10^ M and 10 μL 10^-2^ M R6G aqueous solutions were dropped on the substrates (2×3 mm^2^) and polyvinyl chloride film (π×2.5^2^ mm^2^), respectively. Therefore, the EF was calculated to be 1.41×10^8^, revealing the high SERS activity of the fabricated SERS sensor.

In Figure S13(b), the EF was calculated by the same calculation formula as in Equation (1). In Figure S13(b), 5 μL 10^-10^ M and 10 μL 10^-2^ M R6G aqueous solutions were dropped on the substrates (2×3 mm^2^) and polyvinyl chloride film (π×2.5^2^ mm^2^), respectively. The EF was calculated to be 1.68×10^8^.

**Part 4**

**References**

[1] J. Cai, J. Huang, M. Ge, J. Iocozzia, Z. Lin, K.Q. Zhang, Y. Lai, Immobilization of Pt nanoparticles via rapid and reusable electropolymerization of dopamine on TiO_2_ nanotube arrays for reversible SERS substrates and nonenzymatic glucose sensors, Small, 13 (2017) 1604240.

[2] Z. Xie, F. Zhao, S. Zou, F. Zhu, Z. Zhang, W. Wang, TiO_2_ nanorod arrays decorated with Au nanoparticles as sensitive and recyclable SERS substrates, J. Alloy. Compd., 861 (2021) 157999.

[3] S. Xie, K. Lai, C. Gu, T. Jiang, L. Zhou, X. Zheng, X. Shen, J. Han, J. Zhou, Fine fabrication of TiO_2_/MoO_x_ nano-heterojunctions and investigating on the improved charge transfer for SERS application, Mater. Today Nano, 18 (2022) 100179.

[4] C. Wang, Y. Xu, C. Deng, Z. Liu, R. Wang, H. Zhao, Design and preparation of a recyclable microfluidic SERS chip with integrated Au@Ag/TiO_2_ NTs, RSC Adv., 6 (2016) 113115-113122.

[5] J. Dong, J. Huang, A. Wang, G.V. Biesold-McGee, X. Zhang, S. Gao, S. Wang, Y. Lai, Z. Lin, Vertically-aligned Pt-decorated MoS_2_ nanosheets coated on TiO_2_ nanotube arrays enable high-efficiency solar-light energy utilization for photocatalysis and self-cleaning SERS devices, Nano. Energy, 71 (2020) 104579.

[6] M. Zhang, H. Sun, X. Chen, H. Zhou, L. Xiong, W. Chen, Z. Chen, Z. Bao, Y. Wu, The influences of graphene oxide (GO) and plasmonic Ag nanoparticles modification on the SERS sensing performance of TiO_2_ nanosheet arrays, J. Alloy. Compd., 864 (2021) 158189.

[7] X. Zhao, W. Wang, Y. Liang, J. Fu, M. Zhu, H. Shi, S. Lei, C. Tao, Visible-light-driven charge transfer to significantly improve surface-enhanced Raman scattering (SERS) activity of self-cleaning TiO_2_/Au nanowire arrays as highly sensitive and recyclable SERS sensor, Sensor. Actuat. B-Chem., 279 (2019) 313-319.

[8] H. Fang, C.X. Zhang, L. Liu, Y.M. Zhao, H.J. Xu, Recyclable three-dimensional Ag nanoparticle-decorated TiO_2_ nanorod arrays for surface-enhanced Raman scattering, Biosens. Bioelectron., 64 (2015) 434-441.

[9] P. Bansal, N. Bhullar, D. Sud, Studies on photodegradation of malachite green using TiO_2_/ZnO photocatalyst, Desalin. Water Treat., 12 (2009) 108-113.

[10] J. Li, S. Zhang, J. Yang, X. Zheng, Improved SERS sensitivity of TiO_2_ nanorod films by annealing in vacuum, Vacuum, 194 (2021) 110579.

[11] G. Sinha, L.E. Depero, I. Alessandri, Recyclable SERS substrates based on Au-coated ZnO nanorods, ACS Appl. Mater. Inter., 3 (2011) 2557-2563.

[12] L. Jiang, W. Wei, S. Liu, S.A. Haruna, M. Zareef, W. Ahmad, M.M. Hassan, H. Li, Q. Chen, A tailorable and recyclable TiO_2_ NFSF/Ti@Ag NPs SERS substrate fabricated by a facile method and its applications in prohibited fish drugs detection, J. Food Meas. Charact., (2022) 1-9.

[13] Y. Zhao, L. Sun, M. Xi, Q. Feng, C. Jiang, H. Fong, Electrospun TiO_2_ nanofelt surface-decorated with Ag nanoparticles as sensitive and UV-cleanable substrate for surface enhanced Raman scattering, ACS Appl. Mater. Inter., 6 (2014) 5759-5767.
